# Associating Physical and Photocatalytic Properties of Recyclable and Reusable Blast Furnace Dust Waste

**DOI:** 10.3390/ma17040818

**Published:** 2024-02-08

**Authors:** Nayane O. Chaves, Lucas S. Lima, Michael D. S. Monteiro, Raimundo A. L. Sobrinho, Nilson S. Ferreira, Glenda Q. Ramos, Henrique D. da Fonseca Filho, Rosane M. P. B. Oliveira, Robert S. Matos

**Affiliations:** 1 Postgraduate Program in Materials Science and Engineering (P2CEM), Federal University of Sergipe, São Cristovão 49100-000, SE, Brazil; nayaneochaves@academico.ufs.br (N.O.C.); rosaneboliveira@academico.ufs.br (R.M.P.B.O.); 2 Laboratory of Corrosion and Nanotechnology (LCNT), Federal University of Sergipe, São Cristovão 49100-000, SE, Brazil; lsl.lucas@hotmail.com (L.S.L.); michaelquimica96@gmail.com (M.D.S.M.); 3 Department of Chemical Engineering, State University of Santa Cruz, Rod. Jorge Amado, Km 16—Salobrinho, Ilhéus 45662-900, BA, Brazil; ralsobrinho@uesc.br; 4 Department of Physics, Federal University of Sergipe, São Cristovão 49100-000, SE, Brazil; nilson@academico.ufs.br; 5 Centro Multiusuário para Análise de Fenômenos Biomédicos, Universidade do Estado do Amazonas, Manaus 69410-000, AM, Brazil; gq.ramos@hotmail.com; 6 Laboratory of Synthesis of Nanomaterials and Nanoscopy (LSNN), Physics Department, Federal University of Amazonas-UFAM, Manaus 69077-000, AM, Brazil; hdffilho@ufam.edu.br; 7 Amazonian Materials Group, Federal University of Amapá (UNIFAP), Macapá 68911-477, AP, Brazil

**Keywords:** blast furnace dust, nanoparticles, structural defects, photocatalytic activity

## Abstract

Blast furnace dust waste (BFDW) proved efficient as a photocatalyst for the decolorization of methylene blue (MB) dye in water. Structural analysis unequivocally identified α-Fe_2_O_3_ as the predominant phase, constituting approximately 92%, with a porous surface showcasing unique 10–30 nm agglomerated nanoparticles. Chemical and thermal analyses indicated surface-bound water and carbonate molecules, with the main phase’s thermal stability up to 900 °C. Electrical conductivity analysis revealed charge transfer resistance values of 616.4 Ω and electrode resistance of 47.8 Ω. The Mott-Schottky analysis identified α-Fe_2_O_3_ as an n-type semiconductor with a flat band potential of 0.181 V vs. Ag/AgCl and a donor density of 1.45 × 10^15^ cm^−3^. The 2.2 eV optical bandgap and luminescence stem from α-Fe_2_O_3_ and weak ferromagnetism arises from structural defects and surface effects. With a 74% photocatalytic efficiency, stable through three photodegradation cycles, BFDW outperforms comparable waste materials in MB degradation mediated by visible light. The elemental trapping experiment exposed hydroxyl radicals (OH•) and superoxide anions (O2−•) as the primary species in the photodegradation process. Consequently, iron oxide-based BFDW emerges as an environmentally friendly alternative for wastewater treatment, underscoring the pivotal role of its unique physical properties in the photocatalytic process.

## 1. Introduction

Blast furnace dust waste (BFDW) is a byproduct produced within the blast furnace during cast iron production [1]. The process involves utilizing diverse raw materials such as metallic filler (iron ore, sinter, and pellets), solid fuel (coke or charcoal), and fluxes (dolomite and limestone) [2]. Hence, the chemical composition of BFDW exhibits considerable variability [3], directly linked to the specific raw material employed in the blast furnace alimentation [1]. Generally, BFDW comprises iron oxides (α-Fe_2_O_3_) with trace amounts of other oxides [4,5]. Cast iron production typically generates an average amount of 70–110 kg of BFDW per ton of steel [4]. A significant amount of this byproduct is disposed of in landfills, resulting in a large occupation of landfill volume [6].

It is clear that a suitable eco-friendly use of BWFD has not been explored in the existing literature. Despite that, there is a promising avenue to consider this waste as a photocatalyst, primarily due to its predominant composition of hematite (α-Fe_2_O_3_), a known low optical bandgap (2.3 eV) iron oxide [7,8]. α-Fe_2_O_3_ is a naturally abundant and cost-effective material, as iron is one of the most abundant elements on earth [9]. This abundance contributes to the feasibility and scalability of using α-Fe_2_O_3_-based materials in practical applications. α-Fe_2_O_3_ has shown some photocatalytic activity under visible light irradiation, making it a promising candidate for environmental remediation and wastewater treatment [10,11]. Its ability to harness visible light, which constitutes a significant portion of solar radiation, enhances its practicality for solar-riven applications [12,13]. α-Fe_2_O_3_ exhibits good chemical stability [10], ensuring its performance and structural integrity in various environmental conditions. This stability is crucial for long-term applications, particularly in photocatalytic processes where the material is exposed to reactive species and harsh conditions [14,15,16]. Furthermore, α-Fe_2_O_3_ is an inherently magnetic material [14,17], which is a remarkable attribute for its application in advanced oxidative processes [18]. Notably, the physical properties like particle size, particle shape, and structural defects of nanostructured α-Fe_2_O_3_ contribute to its distinctive magnetic and optical characteristics, enhancing its suitability as a photocatalyst [10,19]. Additionally, the environmentally benign nature of α-Fe_2_O_3_ and its non-toxicity and abundance contribute to its appeal in applications focused on sustainable and green technologies, aligning with the growing emphasis on eco-friendly materials. Moreover, α-Fe_2_O_3_ can be easily modified or functionalized to enhance its properties or tailor it for specific applications, e.g., gas sensor [20], photocatalysis [21], energy storage [22], biomedical applications [23,24], magnetic materials [25], and electrochemical capacitors [22,26]. The introduction of additional components, such as metals or other semiconductors, allows for tuning its electronic structure and optimizing its performance [27]. Thus, the advantages of using materials composed of α-Fe_2_O_3_ lie in their abundance, cost-effectiveness, photocatalytic activity, chemical stability, biocompatibility, magnetic properties, environmental friendliness, and versatility in modification. These attributes make α-Fe_2_O_3_-based BFD waste a versatile and promising material in various scientific and technological fields. However, there is a gap in understanding the intricate relationship between the physical and photocatalytic properties of α-Fe_2_O_3_-based BFDW material, which has remained unexplored thus far.

Several studies have directed their focus toward repurposing BFDW for some viable applications, e.g., civil construction [28,29], adsorption processes [3,30], briquette production [7,31], and wastewater treatment [5,32]. Recently, Guo et al. [33] employed BFDW in producing Fe–C composite electrodes and demonstrated an impressive 95.8% indigo dye removal. Despite extensive efforts to explore alternatives for BFDW reuse, particularly in wastewater treatment, there is a notable absence of reports on its direct application as a photocatalyst for the degradation of AZO dyes. AZO dyes, complex aromatic substances extensively utilized by the paper, cosmetics, textile, pharmaceutical, and food industries [34,35], pose significant environmental challenges. Introducing these compounds into water bodies hinders sunlight penetration and disrupts the photosynthetic activity of the medium, leading to the deterioration of water quality and adverse effects on aquatic flora and fauna [36]. In this regard, removing AZO dyes from wastewater using non-harmful waste materials emerges as a safe and effective approach to enhance water body quality. Among wastewater treatment methods, heterogeneous photocatalysis stands out for its low cost and eco-friendly approaches [37,38]. This method utilizes reactive chemical species such as hydroxyl radicals (OH•) and superoxide anions (O2−•) [39] generated on the surface of semiconductor materials to degrade pollutants, e.g., dyes [40,41], pharmaceuticals [42,43], and pesticides [44,45].

Herein, we present a comprehensive and detailed analysis of the physical and photocatalytic properties of fresh BFDW powder. Our investigation involved employing a range of characterization techniques, enabling the elucidation of its chemical, structural, morphological, optical, and magnetic characteristics. Our main goal is to establish a correlation between the physical and photocatalytic characteristics of the waste. This approach contributes to a deeper understanding of the waste’s properties and allows us to unveil its potential as a photocatalyst for removing methylene blue (MB), a known hazardous AZO dye.

## 2. Materials and Methods

### 2.1. Raw Materials

The blast furnace dust waste (BFDW), whose composition is displayed in Table 1, was supplied by Siderúrgica Norte Brasil S.A (Marabá, Pará, Brazil). Before its use, the waste was filtered through a sieve to obtain particles smaller than 100 mesh. The AZO methylene blue dye (C_16_H_18_ClN_3_S∙H_2_O) (Neon P.A., 319.85 g∙mol^−1^, purity ≥ 99%, Suzano, Brazil) was used in the photocatalytic assays. Furthermore, isopropanol (IPA) (Sigma-Aldrich, ≥99.5%, St. Louis, MO, USA), benzoquinone (BQ) (Sigma-Aldrich, ≥98%,), and disodium ethylenediaminetetraacetate dihydrate (EDTA) (Sigma-Aldrich, ≥99%,) were used as scavengers in the elemental trapping analysis.

### 2.2. Characterization of the BFWD Powder

The chemical functional groups present on BFDW were mapped by a Fourier transform infrared spectrometer (FTIR) (PerkinElmer Spectrum Two™, PerkinElmer, Shelton, CT, USA) using KBr pellets, operating at 4000–400 cm^−1^ and a resolution of 16 cm^−1^. The chemical analysis of BFDW was further performed using a Shimadzu EDX Rayny 720 (Shimadzu, Kyoto, Japan), using an Rh anode as a target, a voltage of 50 kV, and a Si/Li detector. The thermal behavior of BFDW was analyzed in a thermogravimeter Shimadzu TGA-50 (Shimadzu, Kyoto, Japan), working at temperatures of 25–1000 °C and a scan rate of 10 °C∙min^−1^. The structure of BFDW was evaluated using a Shimadzu LabX XRD-6000 diffractometer operating (Shimadzu, Kyoto, Japan) with a Cu X-ray tube (Kα1 = 1.54184 Å). The surface morphology of BFDW was studied using a field emission gun scanning electron microscope (FESEM) Jeol JSM IT500-HR (JEOL Ltd., Tokyo, Japan), operating at 5 kV. Additionally, the particle’s shape and composition of BFDW were investigated in a transmission electron microscope (TEM) Jeol JEM-1400Flash (JEOL Ltd., Tokyo, Japan), operating at 120 kV and equipped with energy-dispersive X-ray spectroscopy (EDX). The N_2_ adsorption–desorption analysis at 77.4 K was performed using a Quantachrome NOVAtouch 1200e analyzer (Anton Paar QuantaTec Inc., Boynton Beach, FL, USA). The Brunauer-Emmett-Teller (BET) method was employed to determine the surface area, while the Dubinin-Astakhov (DA) method was used to ascertain pore volume and size. Before the analysis, the sample underwent a degassing process at 101 °C for 3 h under vacuum. The optical absorption and structural defects of BFDW were examined by diffuse reflectance ultraviolet spectroscopy (DRUV) and photoluminescence (PL), using the Ocean Optics HR2000 (Ocean Insight, Orlando, FL, USA) and Jasco FP-8600 spectrophotometer (JASCO, Tokyo, Japan), working from 200 to 800 nm. The magnetic properties of BFDW were investigated by vibrating sample magnetometry (VSM) obtained in a Lakeshore 7407 magnetometer (Lake Shore Cryotronics, Westerville, OH, USA). The photoelectrochemical (PEC) analysis was conducted under visible light conditions (7.74 W/m^2^). Measurements were performed employing an AUTOLAB potentiostat/galvanostat (PGSTAT 100) and the results were evaluated using the NOVA^®^ 2.1.6 software. The Mott-Schottky analysis was conducted at 1 kHz, within the potential range of 1.5 to −1.5 V, using a 1 mol∙L^−1^ NaOH solution. For impedance analysis, a frequency range of 10 kHz to 0.01 Hz was utilized, with an amplitude of 0.01 mV. The electrochemical system consisted of three electrodes: a vitreous carbon working electrode modified with BFDW powder, Pt wire counter electrode, and an Ag/AgCl (Sat., KCl) reference electrode. Finally, the point of zero charge for BFDW was established using the eleven–point method. This approach was done by introducing 20 mg of adsorbent into a 20 mL aqueous solution and adjusting the pH from 2.0 to 12.0 with 0.10 mol∙L^−1^ HCl or NaOH, to identify the surface neutral charge. After 24 h of agitation in a CIENLAB Shaker Incubator (CIENLAB, Campinas, Brazil) at 200 rpm, chemical equilibrium was reached. The final pH was measured with a pH meter (model PHOX P1000, PHOX Suprimentos Científicos, Colombo, Brazil), establishing the point of zero charge.

### 2.3. Photocatalytic Assay

The degradation of MB dye molecules in a water-based solution in the presence of BFDW was studied by a photocatalytic assay. For this purpose, 10 mg of photocatalyst waste was added into 50 mL of the MB solution (10 mg∙L^−1^) and kept under vigorous agitation for 5 min. The suspension was subjected to visible light (7.74 W∙m^−2^) for 180 min using a 600–W halogen lamp. The absorbance of the irradiated solution (3 mL) was measured at λ_max_ = 664 nm using a VARIAN, Cary 100 spectrophotometer (Spectralab Scientific, Markham, ON, Canada). Equations (1) and (2) computed the overall decolorization and degradation kinetics of the MB dye [46], where at t = 0 min (before irradiation), the absorption and equilibrium concentration at λ_max_ = 664 nm are represented by $\gamma_{0}$ and C_0_, respectively. After irradiation at t = 180 min, the absorption and equilibrium concentration at λ_max_ = 664 nm are represented by γ and C, respectively, to obtain the pseudo-first-order rate constant (k). An identical experiment was performed in the dark as a control experiment (blank). Moreover, an elemental trapping experiment (ETE) and a total organic carbon (TOC) test were performed to investigate the chemical species responsible for the degradation of MB molecules and their mineralization efficiency, respectively. The ETE involved using 3 mL of IPA, 0.1 mmol of BQ, and 0.1 mmol of EDTA as scavengers. The TOC test was performed using a thermoreactor Analytik Jena (Jena, Germany), Model NC 3100 to measure the TOC before (To) and after (T) the photocatalytic process as a function of irradiation time. The mineralization efficiency of MB organic molecules was calculated using Equation (3) [46].

(1) $$\%Decolorization=\left(\frac{C_{0}-C}{C_{0}}\right)\times 100\%=\left(\frac{\gamma_{0}-\gamma }{\gamma_{0}}\right)\times 100\%$$

(2) $$kt=-ln\left(\frac{\gamma }{\gamma_{0}}\right)$$

(3) $$Mineralization\;efficiency\;(\%)=\left(1-\frac{T}{T_{0}}\right)\times 100\%$$

## 3. Results and Discussion

### 3.1. Characterization of BFDW Powder

**Figure 1:**
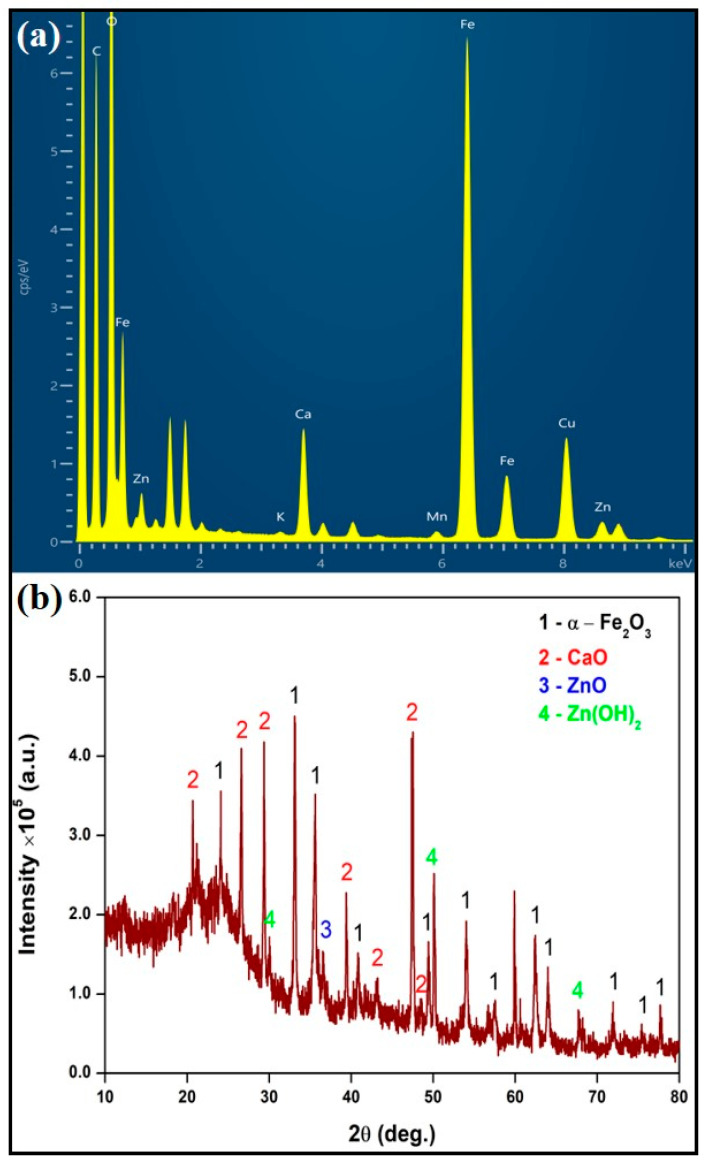
(**a**) EDX spectrum and (**b**) XRD pattern of pure blast furnace dust waste (BFDW).

The compositional analysis of BFDW powder (Table 1) unequivocally demonstrates that the predominant component of BFDW powder is hematite (α-Fe_2_O_3_), comprising approximately 94% of the total composition. This finding aligns seamlessly with our EDX analysis, as illustrated in Figure 1a. It is worth noting that minor quantities of calcium oxide (CaO) (~3%), potassium oxide (K_2_O) (~2%), zinc oxide (ZnO) (0.741%), and manganese oxide (MnO) (0.665%) were also detected. Elemental analysis robustly confirmed the presence of these elements in the composition of BFDW powder, in addition to other minor impurities, e.g., Al, Si, P, Cl, and Ti. Such unique elemental characteristics of BFDW can be ascribed to the composition of the precursor raw materials [47,48], e.g., dolomite [2], quartz [49], sinter constituents [50], and pellets [51].

The structural analysis of BFDW, depicted in Figure 1b, discloses that the waste is primarily composed of α-Fe_2_O_3_ (89.9%), CaO (7.7%), ZnO (1.5%), and Zn(OH)_2_ (0.9%). This finding aligns well with the chemical analysis, except for the absence of other phases. These dominant phases were successfully identified using the ICSD cards PDF#00-017-0912 (CaO), PDF#01-089-0596 (α-Fe_2_O_3_), PDF#01-075-1526 (ZnO), and PDF# 00-041-1359 (Zn(OH)_2_). The CaO phase crystallizes in the cubic structure associated with the Fm$\bar{3}$m space group [52,53], with crystallographic planes (101), (114), (111), (200), (310), and (202) evident. Further, the small ZnO and Zn(OH)_2_ phases present a hexagonal and tetragonal packaging corresponding to the P6_3_mc and I4_1_/amd space groups. The ZnO reflection is assigned to the (101) crystallographic plane observed at 2θ = 36.5°. On the other hand, the Zn(OH)_2_ phase displayed the crystallographic planes (206), (325), and (2410). Finally, the primary phase (α-Fe_2_O_3_) crystallizes in a rhombohedral-trigonal crystallographic structure belonging to the R-3c space group, displaying the crystallographic planes (104), (110), (113), (024), (116), (018), (214), (300), (119), (220), and (036). Furthermore, the prominent broad halo spanning the range of 2θ = 15–30° further indicates that the residue is predominantly influenced by the presence of organic compounds arising from coal combustion and raw materials.

**Figure 2:**
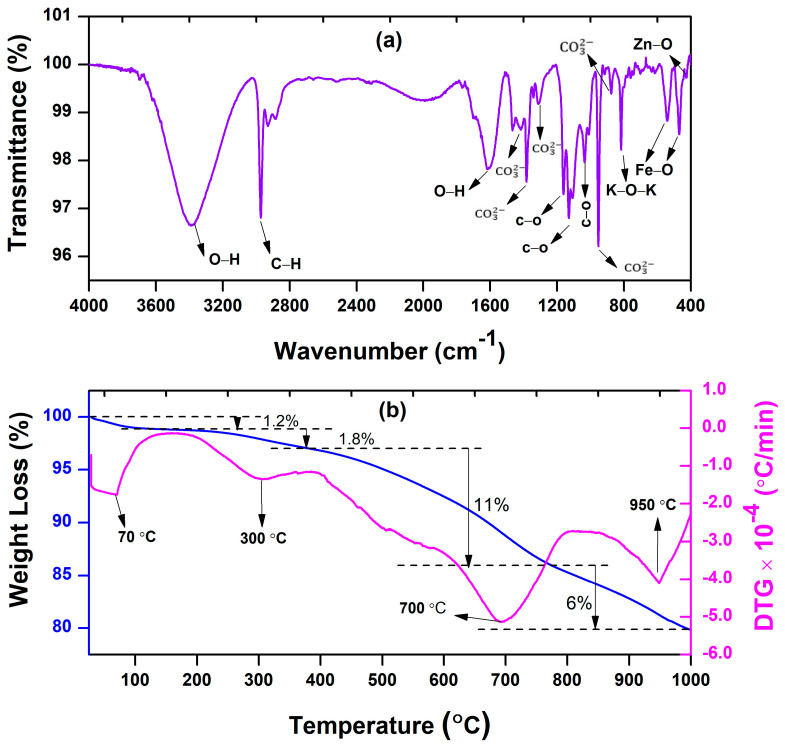
(**a**) FTIR spectrum and (**b**) TGA/DTG curves of pure blast furnace dust waste (BFDW).

The chemical structure of the BFDW powder was also investigated by an FTIR analysis, as illustrated in Figure 2a. The surface-bound water molecules are behind the bands at 3397 and 1625 cm^−1^, which are ascribed to the stretching vibration [54,55] and symmetric angular deformation [56,57] of hydroxyl groups (O−H), respectively. The band at 2975 cm^−1^ is assigned to the C–H stretching vibration associated with alkyl groups [55,58]. The weak and narrow bands at 1420 and 877 cm^−1^ are attributed to the asymmetric stretching and out-of-plane vibrations of carbonate $CO_{3}^{2-}$ [59]. The bands at 1383 and 1305 cm^−1^ confirm the substantial presence of $CO_{3}^{2-}$ molecules [46] adsorbed on the BFDW surface. The distinctive intensity bands at 1160, 1124, and 1031 cm^−1^ can be associated with the aromatic stretching vibrations of C–O [60]. The strong and narrow band at 825 cm^−1^ is linked to the stretching vibration of K–O–K [61], indicating the presence of the K_2_O phase. Further, the well-defined bands at 538 and 468 cm^−1^ arise due to Fe–O stretching vibrations, directly related to the presence of the hematite phase (α-Fe_2_O_3_) [17,62,63]. Finally, the weak band around 427 cm^−1^ is attributed to the stretching vibrations of Zn–O, indicating the presence of ZnO structures in the BFDW powder. These results are well aligned with our chemical analysis, confirming two known photocatalysts α-Fe_2_O_3_ and ZnO. However, it is worth noting that there is a notable presence of surface contamination, mainly from carbonate molecules, corroborating the observations made in the XRD analysis (Figure 1b). According to the existing literature, such behavior arises from the combustion of eucalyptus-based coal and the raw materials used in the furnace reaction during cast iron production.

The existence of organically adsorbed material on the surface of BFDW particles significantly imparts a distinct and unique quality to this waste. This outcome is expected, given that the composition of blast furnace dust waste is highly reliant on the specific raw materials employed in the cast iron manufacturing process, being one of the most complex metallurgical wastes [64]. We confirm such an aspect by examining the thermal stability of BFDW through TGA/DTG analysis, with the results presented in Figure 2b. The thermal decomposition analysis of BFWD reveals that between 25 and 400 °C, there is a small mass loss of 3%, with significant thermal events observed at 70 and 300 °C. The initial thermal event is likely associated with free moisture loss [65], while the subsequent event can be attributed to the release of chemically adsorbed water [66]. The most substantial weight loss of 11%, occurred between 400 and 800 °C, resulting in a broad DTG thermal event centered at 700 °C. Such behavior can be ascribed to the robust liberation of CO_2_ during the decomposition of $CO_{3}^{2-}$ molecules [67,68] present on the BFDW surface. The final stage of decomposition, extending up to 1000 °C, discloses an additional 6% mass reduction, leading to a thermal event at 950 °C, as can be seen in the DTG analysis. This event can be attributed to the conversion of α-Fe_2_O_3_ into wüstite (FeO) [69], a phase that has been previously documented for a BFDW waste [2]. Hence, it is evident that the predominant organic component in BFDW powder consists of carbonates, which can be effectively removed at temperatures above 400 °C. Additionally, there were no observable phase transformations up to 900 °C, indicating the thermal stability of the primary phase (α-Fe_2_O_3_) at lower temperatures.

The results of N_2_ adsorption–desorption analyses, detailing the surface area, volume, and average pore diameter of BFDW, are presented in Table 2. Surface area determination employed the BET method, while the evaluation of pore volume and average diameter utilized the DA method. In terms of pore classification, adsorbents or photocatalysts typically exhibit micropores (average pore diameter < 20 Å), mesopores (average pore diameter between 20 and 500 Å), and macropores (average pore diameter > 500 Å) [70]. Table 2 indicates that the average pore diameter of blast furnace dust is 15.20 Å, classifying it as a microporous material. The N_2_ adsorption and desorption isotherm behavior in Figure S1 aligns with the type IV standard, which IUPAC classifies as adsorption on mesoporous solid materials. However, this contradicts the information obtained by the DA method. This discrepancy arises because the presence of microporous pores can influence the isotherm’s shape, even if they constitute a small proportion of the total pore volume. In such cases, the isotherm may exhibit a shape more similar to type IV, featuring a distinct region of linear adsorption followed by curvature at high pressure [71,72]. Hence, our assessment indicates that the average pore diameter encompasses the combined volume of micropores and mesopores present in the BFDW powder. In the study by Bhuyan et al. [73], a microporous filter was developed using blast furnace waste to remove MB dye, where the surface area played a crucial role in removal efficiency. This suggests that blast furnace dust waste can serve as a viable adsorbent or photocatalyst for the cost–effective removal of organic dye molecules in wastewater treatment processes, primarily due to its substantial surface area (284 m^2^∙g^−1^).

**Figure 3:**
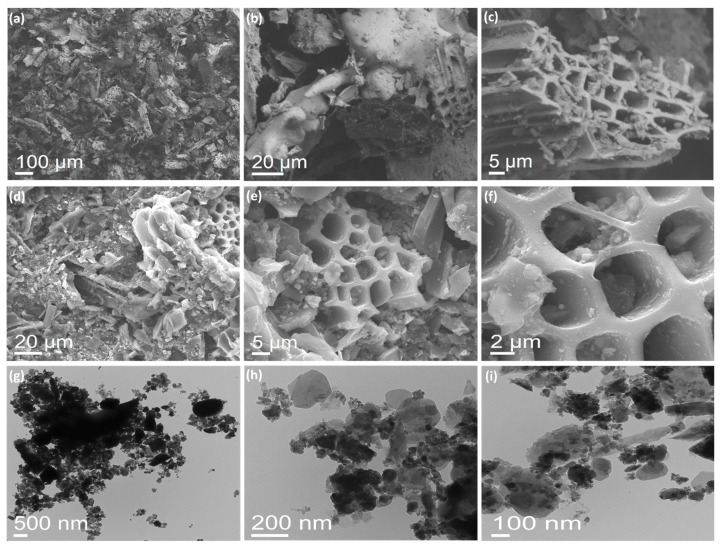
(**a**–**c**) SEM, (**d**–**f**) FESEM, and (**g**–**i**) TEM images of pure blast furnace dust waste (BFDW) obtained under different magnifications.

Our morphological analysis in Figure 3 provides a broad overview of the BFDW powder surface at different scales. SEM images (Figure 3a–c) reveal that the BFDW exhibits a porous surface morphology comprised of structures with diverse shapes and arrangements consistent with the surface porosity analysis. This observation is corroborated by the FESEM analysis (Figure 3d–f), highlighting the microstructure of the BFDW powder that primarily consists of highly porous structures agglomerated and adorned with smaller particles (Figure 3f), forming substantial clusters. In particular, these clusters display distinctive shapes and sizes, likely attributed to the diversity of phases identified in the chemical and structural analysis. TEM analysis (Figure 3g–i) further underscores that the BFDW smallest particles range from 10–30 nm, indicating that the aggregation of nanoparticles essentially forms the powder. Moreover, these nanoparticles exhibit diverse shapes, e.g., nanospheres, nanorods, nanoneedles, and other particles with robustly irregular shapes, suggesting that there is a different anisotropic growth of the particles in the blast furnace atmosphere due to the combustion of raw materials. Our FTIR analysis detected a notable presence of organic matter within the powder structure (Figure 2a). These compounds or functional chemical groups function as capping agents, facilitating the agglomeration of smaller particles, which leads to the formation of larger structures, as prominently observed in our morphological analysis. In fact, capping agents play a pivotal role in promoting particle agglomeration, resulting in the formation of nanoparticles with distinctive size, shape, and optical properties, in accordance with the existing literature [74,75,76,77].

**Figure 4:**
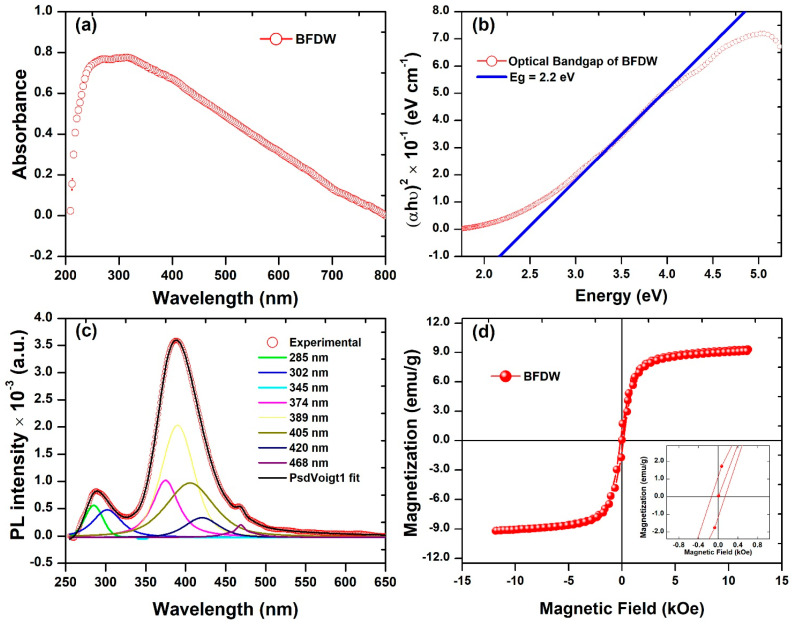
(**a**) Optical absorption spectrum, (**b**) Tauc plot (direct gap), (**c**) Deconvoluted photoluminescence spectrum, and (**d**) Magnetic hysteresis curve of BFD waste. The magnified insertion in Figure 3d displays the critical region for remanent magnetization and coercivity estimation.

The distinctive structural and morphological characteristics of BFDW undoubtedly underlie its optical properties, as depicted in Figure 4. In this regard, a broad UV-Vis absorption band was observed from the BFDW solution (Figure 4a), and its appearance is similar to that reported for other distinctive-shaped α-Fe_2_O_3_ nanoparticles [78,79]. Nevertheless, this broad spectrum underwent deconvolution using a Gaussian function (Figure S2), exposing the presence of three sub-bands centered at 297, 370, and 527 nm. The first band is associated with the metal-to-ligand charge transfer properties of α-Fe_2_O_3_ [80]. The subsequent bands can be ascribed to A1→6E6 and A1→62(T14) transitions in the α-Fe_2_O_3_ crystal [80]. However, these bands can also be attributed to the monodisperse nature and the transition of O2p → Zn3d within ZnO crystals [57,81], respectively. The optical bandgap of BFWD (Figure 4b) was determined to be 2.2 eV, which closely aligns with the value reported for α-Fe_2_O_3_ bulk [82,83], confirming that the optical properties of BFDW are primarily due to this phase. Indeed, as evident from our chemical and structural analysis, the waste powder is predominantly composed of α-Fe_2_O_3_ particles, indicating that the optical properties of BFWD are mainly assigned to this phase.

The existing literature establishes that hematite bulk does not exhibit photoluminescent properties [84,85,86], suggesting that any optical property associated with α-Fe_2_O_3_ particles present in BFDW must be likely linked to effects stemming from their nanostructured nature. These effects may include quantum phenomena, forbidden d-d transitions, magnetic relaxations, and cation-cation resonant energy transfers [87,88], which can influence the material’s properties. In fact, the deconvoluted PL spectrum (Figure 4c), acquired under excitation at 260 nm, unveils the presence of several structural defects within the α-Fe_2_O_3_ lattice, as summarized in Table 3. The emissions at 285 nm (4.35 eV), 302 nm (4.10 eV), and 345 nm (3.59 eV) are ascribed to the mid-band-edge (MBE) UV absorption and are associated with the transitions from a fundamental ground state ($t_{1g}$) to a higher-energy excited state ($e_{g}$) between the d orbitals. The luminescence peak at 345 nm (3.59 eV) can also be attributed to MBE or the compensation of oxygen vacancies (V_O_) [89]. The emission observed at 374 nm (3.32 eV) is a result of exciton emissions. This luminescence effect stems from electronic transitions, with the smallest exciton state originating from excitation between the VB (derived from a combination of ${Fe}_{3d}$ and $O_{2p}$ states) and CB (primarily derived from ${Fe}_{4s}$) [90]. The peak at 389 nm (3.19 eV) is assigned to the near-band-edge (NBE) UV emission or due to the recombination of holes (h^+^) generated by photons with a specific defect charge state. The emissions at 405 nm (3.06 eV) and 420 nm (2.95 eV) are associated with deep-level emissions, which originate from oxygen interstitial (O_i_) and oxygen vacancies (V_O_) for both ZnO and α-Fe_2_O_3_ crystals [91,92,93]. The blue emission at 468 nm (2.65 eV) is assigned to V_O_ defects in ZnO crystals [94] or due to the ^6^A_1g_ → ^4^T_1g_ and d-d transitions of Fe^3+^ ions, which result from the crystal field splitting of a FeO_6_ octahedron with O_h_ symmetry as a first approximation in the α-Fe_2_O_3_ crystal [84]. Hence, the lattice of α-Fe_2_O_3_ and ZnO crystals found in the BFDW powder are primarily composed of O_i_ and V_O_ defects, which can explain any photocatalytic property of BFDW.

Additionally, defects in hematite particles and their nanostructured behavior confer magnetic properties to the BFDW powder, as evidenced in Figure 4d. Noticeably, our room temperature (298 K) VSM measurement revealed that BFDW displays a weak ferromagnetic behavior, which can be attributed to structural defects in α-Fe_2_O_3_. The saturation magnetization (Ms), remanent magnetization (M_R_), and coercivity (H_C_) were found to be 9.41 emu/g, 1.15 emu/g, and 0.02 kOe, respectively, indicating soft magnetic properties [101]. It is known that α-Fe_2_O_3_ possesses a crystalline lattice with Fe^3+^ ions arranged in layers, where oxygen atoms are situated between these layers. Hence, the presence of O_i_ defects can influence the magnetic interactions among Fe^3+^ ions, giving rise to unique magnetic properties. Similarly, Vo defects can introduce local distortions in the magnetic structure, impacting the magnetic interactions between Fe^3+^ ions by introducing small polarons strongly bound to the positively charged defect centers [102,103]. The structural defects can also influence the Morin transition and other aspects of the magnetic transition [104], resulting in the intriguing magnetic properties of α-Fe_2_O_3_. Furthermore, our morphological analysis revealed the presence of nanoparticles with sizes ranging from 10 to 30 nm. These smaller particles can also have an impact on the magnetic properties of BFDW, as smaller particles exhibit a higher surface-to-volume ratio [105]. This can give rise to distinctive surface magnetic behavior, where surface magnetic effects dominate the overall magnetic properties. The presence of magnetic properties in BFDW is interesting as it renders the waste recoverable and suitable for reuse in photocatalytic applications, which was extensively explored in our comprehensive photocatalytic tests.

**Figure 5:**
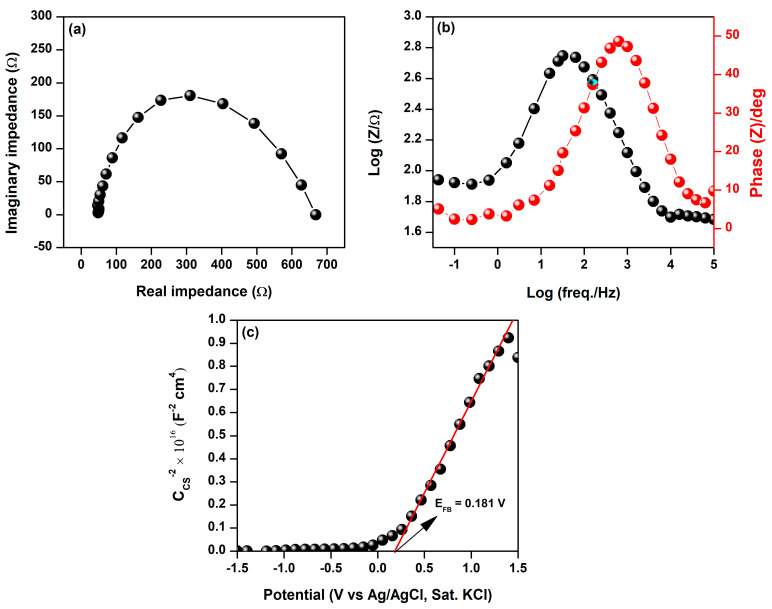
(**a**) Nyquist, (**b**) Bode mode and phase, and (**c**) Mott–Schottky analyses of BFD waste obtained under visible light conditions.

We also investigated the electrical conductivity performance of BFDW. Photocurrent densities were measured under visible light conditions (7.74 W∙m^−2^). We employed EIS analysis to scrutinize the charge transfer kinetics at the electrode–electrolyte interface, and the outcomes are illustrated in Figure 5. Figure 5a depicts the Nyquist plot of the BFDW sample, which displays electrode resistance (R_p_) and charge transfer resistance (R_ct_) values of 47.8 and 616.4 Ω, respectively. It is known that most semiconductor oxides typically exhibit low electrical conductivity [106]. Hence, the α-Fe_2_O_3_-based BFD waste is expected not to demonstrate high charge carrier mobility at the electrode–electrolyte interface, as evidenced by the elevated R_ct_ value. The reported value is notably higher than the maximum reported by Sarma et al. [107] for a dark condition in a doped α-Fe_2_O_3_ system (358.2 Ω). However, it remains considerably lower than the values documented by Niu et al. [108] (1735–90,000 Ω) obtained for varied potentials (0.9–1.4 V) under visible light conditions. The Bode impedance and phase curves of BFD waste are shown in Figure 5b. In the lower frequency region, there is a slight decrease in impedance as the applied frequency increases. Conversely, in the higher frequency range, a significant reduction in impedance becomes evident. Further, the correlation between |Z| and frequency follows a nearly constant trend within the mid-frequency range, indicating quasi-independent behavior. This suggests that ionic diffusion from the aqueous electrolyte to BFD waste remains consistent across varying frequencies. In this regard, the resistance to diffusion of OH ions in the liquid electrolyte can be ascribed to an elongated pathway between the Pt electrode and the interface of BFDW. Thus, the impedance-phase plots illustrate the BFD waste’s charge transport and recombination processes. Moreover, the phase angles ranging from 0–50° indicate a robust capacitive behavior [109], which can be a remarkable characteristic of the photocatalytic performance of BFDW. Notably, the high capacitive behavior can exert considerable influence, facilitating efficient charge transfer, boosting reactivity, and providing the potential for rapid redox reactions.

We conducted additional investigations into the band structures of BFDW using Mott–Schottky (M–S) analysis. Mott–Schottky analysis offers a straightforward and accurate way to extract information about the semiconductor properties and charge carriers at the interface of a semiconductor–electrolyte. Our M–S analysis performed at 1 kHz is shown in Figure 5c and was used to investigate the flat band potential (E_fb_ in V) and donor density (N_d_) of the BFDW photocatalyst. The E_fb_ and N_d_ were estimated using the Mott−Schottky theory, according to the Equations (4) and (5) [110].

(4) $$\frac{1}{C_{sc}}=\frac{2}{q\epsilon \varepsilon_{0}\sigma^{2}N_{d}}\left(E-E_{fb}-\frac{k_{B}T}{q}\right)$$

(5) $$N_{d}=\frac{2}{q\epsilon \varepsilon \sigma^{2}\mu }$$

where $C_{sc}$ denotes the space–charge capacitance, q represents the elementary charge, ϵ is the dielectric constant of the semiconductor, $\varepsilon_{0}$ is the permittivity of free space, σ is the electrode area, V is the applied potential, $k_{B}$ is the Boltzmann constant, T is the temperature in degrees Kelvin, and μ is the slope of the Mott–Schottky plot at $\frac{1}{C_{cs}^{2}}=0$. The energy difference (δE) between the conduction band (E_CB_) and the Fermi level ($E_{FL}$) was further calculated using Equation (6).

(6) $$\delta E=E_{CB}-E_{FL}=k_{B}Tln\left(\frac{N_{d}}{N_{v}}\right)$$

where Nv=22πµ0kBh232. In this equation, ${µ}_{0}$ and h are the electron effective mass and Plank constant, respectively. Finally, the energy values of the Fermi level ($E_{FL}$), valence band (E_VB_), and conduction band (E_CB_) in eV were determined by converting the Fermi level potential E_F_ (V vs. SHE) in the vacuum scale using Equations (7)–(9) [111].

(7) $$E_{FL}(eV)={-E}_{FL}\left(V\;vs.SHE\right)-4.44\left(V\right)$$

(8) $$E_{CB}\left(eV\right)=E_{FL}\left(eV\right)+0.3\left(eV\right)$$

(9) $$E_{VB}(eV)=E_{CB}\left(eV\right)-E_{g}(eV)$$

The linear extrapolation showed that the slope of the Mott–Schottky curve is positive, indicating that BFDW is dominated by an α-Fe_2_O_3_ n–type semiconductor. At $\frac{1}{C_{cs}^{2}}=0$, the $E_{fb}$ value was found to be 0.181 (V vs. Ag/AgCl), corresponding to 0.378 (V vs. SHE). The $N_{d}$ and the difference δE values were computed as 1.45 × 10^15^ cm^−3^ and 0.225 eV, respectively. The corresponding values of E_CB_ and E_VB_ in V are 0.612 and −1.588, respectively. By employing Equations (7)–(9), the values of the band structures of BFDW in eV were estimated to be $E_{FL}=-4.818\;eV$ and $E_{CB}=-4.593\;eV$, and $E_{VB}=-6.793\;eV$. The n-type conductive properties of α-Fe_2_O_3_ structures were also previously reported by Li et al. [112]. The flat-band potentials and donor densities of their α-Fe_2_O_3_ samples under dark and light conditions were influenced by morphological differences. In contrast, Wodka et al. [113] observed characteristic steps in the M–S curve for an α-Fe_2_O_3_/TiO_2_ composite, associating them with surface states and demonstrating a significant effect on the flat-band potential due to the presence of α-Fe_2_O_3_. However, the BFDW, primarily composed of α-Fe_2_O_3_, exhibits a positive M–S slope without such steps, indicating a different behavior. Thus, the M–S analysis of BFDW sheds light on its semiconductor properties, showcasing distinct features compared to other studies. The n-type behavior and specific band structure values contribute to understanding BFDW’s potential for applications in photocatalysis, with its recoverable and reusable attributes highlighted.

Before assessing the photocatalytic activity of BFDW, the zero-point charge (ZPC) was examined to confirm the potential for dye adsorption on the photocatalyst’s surface. It is known that photocatalysts or adsorbent materials acquire a surface charge by dissociating their surface functional groups [114]. In this regard, the prevalence of positive or negative charges depends on the pH of the solution. The point at which the net charge on the adsorbent surface equals zero, indicating an equilibrium between the number of opposite charges, is referred to as ZPC. Below the ZPC, the surface charge is positively charged, favoring the adsorption of anions; otherwise, it is negatively charged, facilitating the adsorption of cations [115]. The ZPC of BFDW was determined to be 7.26, as shown in Figure S3. Thus, for the present experimental conditions, the adsorption of MB dye is likely favorable, which was confirmed by our photocatalytic experiments extensively described below.

### 3.2. Photocatalytic Activity of BFDW

**Figure 6:**
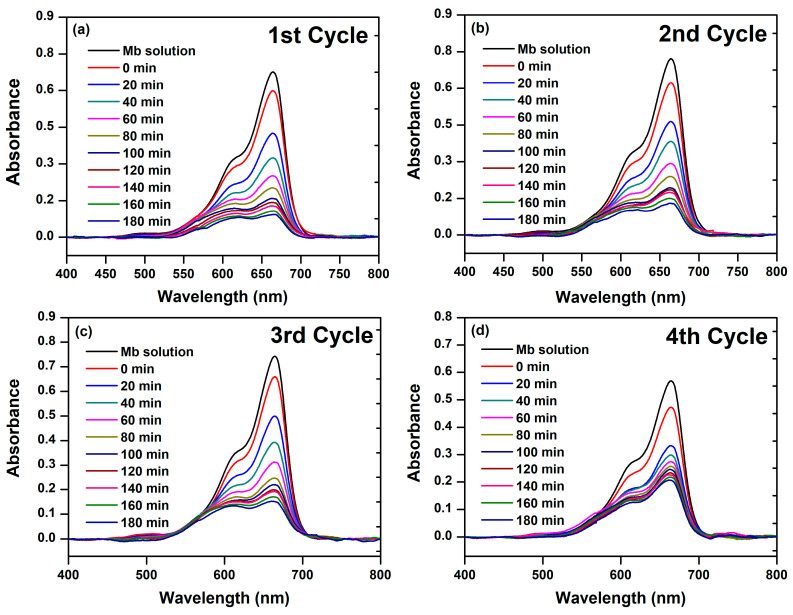
UV-Vis spectra of MB dye degradation in aqueous solutions containing ferromagnetic BFD waste and irradiated with visible light for 180 min, obtained after (**a**) 1st, (**b**) 2nd, (**c**) 3rd, and (**d**) 4th cycles of reuse.

The spotlight on harnessing defective nanostructured systems for mediating photocatalytic processes has risen sharply, fueled by their versatility and positive impact in diverse scenarios [116]. Whether it is purifying water, enhancing air quality, or skillfully eliminating pollutants, the efficiency and sustainability of this technology have garnered widespread acclaim [117]. The pursuit of eco-friendly and innovative solutions propels the ongoing exploration of nanoparticle photocatalysis, cementing its role as a crucial tool in the modern scientific and technological landscape. Herein, we conducted our photocatalytic assay utilizing nanostructured BFDW as a photocatalyst. Firstly, leveraging the magnetic properties of α-Fe_2_O_3_ particles within BFDW, we could recover and reuse the waste for up to 4 cycles in the photocatalytic process. The assessment involved monitoring UV-Vis absorption curves of MB dye molecules at 664 nm over various time intervals, as displayed in Figure 6. Prior to this procedure, a solution containing BFDW was kept in the dark, and the characteristics of the MB band at 664 nm were analyzed for 180 min (Figure S4). In this experiment, a slight reduction in the MB band at 664 nm can be observed after 180 min, likely attributed to the adsorption of organic dye molecules on the BFDW surface. On the other hand, as evident in Figure 6a–c, the maximum absorption of MB dye consistently and similarly decreased from 0 to 180 min in the 1st, 2nd, and 3rd cycles, indicating persistent and similar destruction of the organic dye molecules of MB. However, in the 4th cycle (Figure 6d), this decrease is not significant, suggesting that BFDW is better recoverable and reusable for up to three cycles in the photocatalytic process.

**Figure 7:**
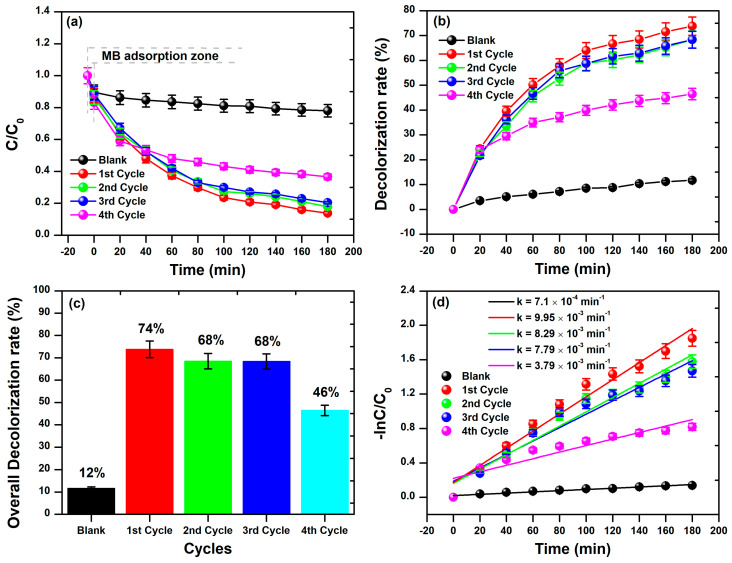
(**a**) The C/C_0_ factor, (**b**) Decolorization rate, (**c**) Overall decolorization rate, and (**d**) First-order kinetics of MB dye degradation based on cyclic degradation experiment.

Figure 7 illustrates a more in-depth examination of the photocatalytic performance of BFD waste in an aqueous solution against MB dye molecules. Our analysis was based on the UV-Vis spectra obtained in triplicate for each cycle. By keeping the MB dye solution with 10 mg of BFDW in darkness for 30 min, the MB adsorption rates were computed as 8 ± 1.5% (1st cycle), 10 ± 1% (2nd cycle), 9.5 ± 1.6% (3rd cycle), and 11 ± 2.1% (4th cycle). The elevated adsorption rate of MB molecules on the BFDW surface (>10%) can be notably attributed to the remarkably porous microstructure of the powder, as depicted in Figure 3. The irradiation of the MB dye solution with visible light revealed variations in the photocatalysis of MB molecules under different degradation cycles, as shown in Figure 7. Notably, the average C/C_0_ factor (Figure 7a) consistently decreased across all cycles compared to the dark experiment (Blank). Despite this trend, a comparative examination revealed that the behavior of C/C_0_ was nearly identical from the 1st to the 3rd cycle, whereas the 4th cycle exhibited a distinct and less pronounced reduction of C/C_0_. Similarly, the decolorization rate of the MB dye remained quite consistent for the 1st, 2nd, and 3rd cycles, with a decrease observed in the 4th cycle (Figure 7b). Figure 7c also illustrates that there was no statistically significant difference in the overall decolorization rates from the 1st to the 3rd cycle, calculated as 74% (1st cycle), 68% (2nd cycle), and 68% (3rd cycle). These rates substantially exceeded the decolorization rate found in the dark experiment (12%), providing suitable evidence that the destruction of MB hazardous molecules is activated by the physical properties of the particles present in the BFDW powder, mainly α-Fe_2_O_3_ nanoparticles. In contrast, we computed that the rates of MB adsorption on the waste surface did not surpass ~13%, indicating that the decolorization rate computed in the dark experiment primarily reflects the adsorption of dye molecules on the MB particles’ surface rather than their destruction. Hence, in addition to showcasing the highest decolorization rate of MB molecules after 180 min of visible light exposure, the 1st cycle also demonstrated the highest rate of the photocatalytic reaction (Figure 7d) (9.95 × 10^−3^ min^−1^), followed closely by the 2nd (8.29 × 10^−3^ min^−1^) and 3rd (7.79 × 10^−3^ min^−1^) cycles. This observation means that the photocatalytic process unfolds comparably from the 1st to the 3rd cycle. As a result, the analysis of the photocatalytic process aligns with the visually observed behavior in the UV-Vis curves, substantiating that the BFDW powder can be recycled and reused for up to 3 cycles without a significant loss of photocatalytic performance.

**Figure 8:**
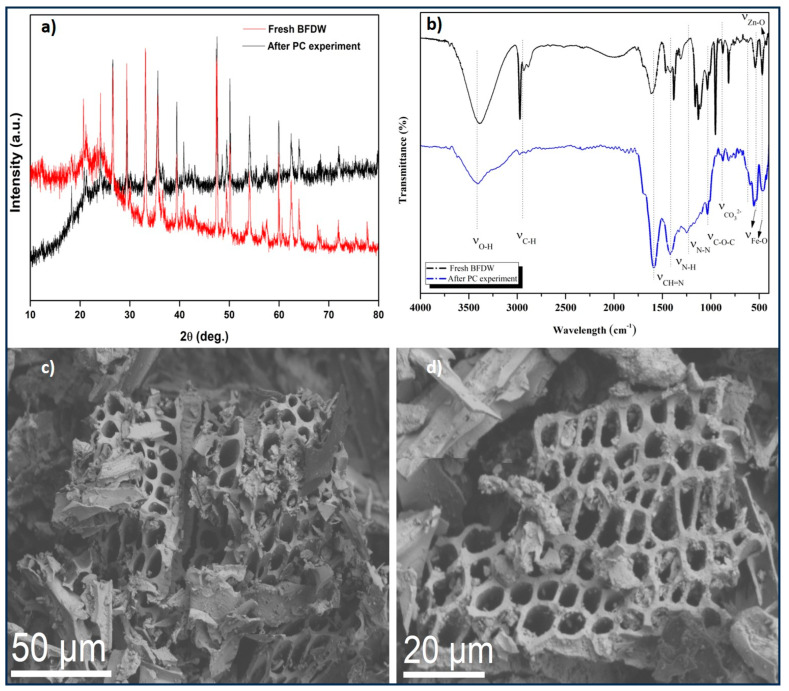
(**a**) Comparison of the XRD patterns obtained before and after the photocatalytic experiment, (**b**) Comparison between the FTIR curves before and after the photocatalytic experiment, and (**c**,**d**) SEM images of BFDW after the photocatalytic experiment, obtained under different magnifications.

We also scrutinized the morpho-structural characteristics of BFDW after the photocatalytic experiment, as illustrated in Figure 8. Figure 8a presents a comparison between the XRD pattern obtained before and after the experiment, indicating that no significant structural changes occurred post-photocatalytic experiment. However, Figure 8b showcases a comparison between the FTIR curves before and after the photocatalytic experiment. Notably, an increase in the intensity of the CH=N bands at 1594 cm^−1^, N–H at 1420 cm^−1^, and N–N at 1240 cm^−1^ was observed after the photocatalytic process, suggesting a greater presence of nitrogen functional groups in BFDW, attributable to the adsorption of MB on its surface [118]. At 1382 cm^−1^, symmetric C–O–C stretching vibrations are noted; at 1131 cm^−1^, the C–O band is observed. The band at 1430 cm^−1^ corresponds to the asymmetric stretching and out-of-plane vibration of $CO_{3}^{2-}$ molecules [55]. Additionally, bands at 540 cm^−1^ and 471 cm^−1^ are assigned to the stretching vibration Fe–O, indicative of α-Fe_2_O_3_ ceramics [55]. Furthermore, the band at 428 cm^−1^ is associated with the Zn–O bending vibration, reflecting the presence of small amounts of ZnO compound. The differences in the chemical structure observed in this analysis are attributed solely to the presence of MB dye functional groups adsorbed on the photocatalyst’s surface. Moreover, Figure 8c,d, obtained after the photocatalyst procedure, reveals that the microstructure of BFDW remains unchanged. The observed morphology is well aligned with the morphological analysis of the pristine BFDW powder, as demonstrated in Figure 3a–c. Thus, our results prove that the morpho-structural characteristics of BFDW remain unchanged after the photocatalytic tests.

The physical characteristics of semiconductor materials, including particle size, shape, surface area, and structural defects, are widely acknowledged as pivotal factors influencing their photocatalytic properties [119,120]. Our analysis revealed that α-Fe_2_O_3_ and ZnO constitute the main defective phases, comprising approximately 90–92% of the BFDW composition. This indicates that the photocatalytic activity of the residue can be predominantly attributed to these phases. Additionally, our observations identified particles with diverse shapes and sizes, and in particular, a significant portion falling within the 10–30 nm range, contributing to a high surface-to-volume ratio of BFDW powder. Despite previous reports suggesting that α-Fe_2_O_3_ may not be an efficient photocatalyst due to its low optical absorption [10,87], our study demonstrated that BFDW exhibited good optical absorption. This observation is partly attributed to the presence of ZnO, a semiconductor known for its ability to absorb visible light. Such observation allowed us to identify some structural defects, primarily O_i_ and Vo, native anionic defects commonly found in nanostructured α-Fe_2_O_3_ and ZnO [93,121]. The existing literature underscores the crucial role of these defects in generating reactive chemical species capable of degrading synthetic dye molecules [122]. On this point, these defects contribute to delaying the recombination of charge carriers, thereby enhancing the production of reactive species such as (OH•) and (O2−•), responsible for dye degradation [123,124,125]. These findings show that the photocatalytic activity of BFDW is mainly attributed to the physical properties of ZnO and α-Fe_2_O_3_, which was further demonstrated to exhibit magnetic behavior, facilitating its recovery and consistent reuse for up to 3 degradation cycles. 

**Figure 9:**
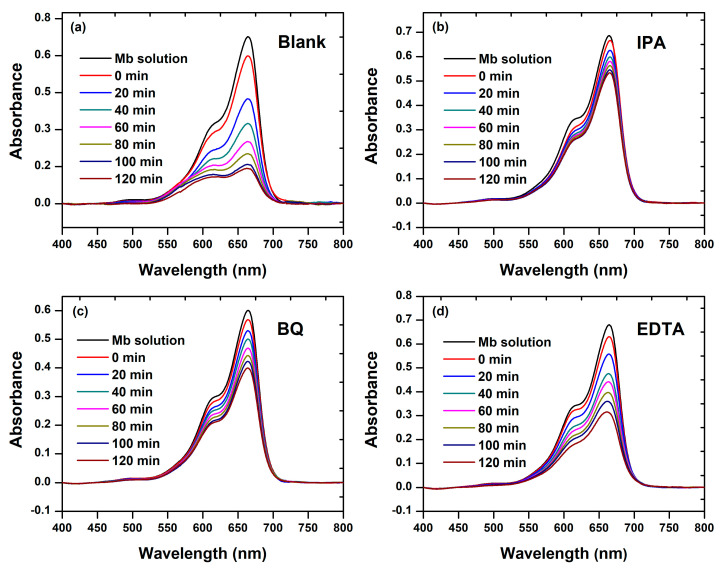
UV-Vis spectra of MB dye degradation in aqueous solutions containing ferromagnetic BFD waste and irradiated with visible light for 120 min, obtained under the action of (**a**) No scavenger, (**b**) IPA, (**c**) BQ, and (**d**) EDTA as scavengers.

**Figure 10:**
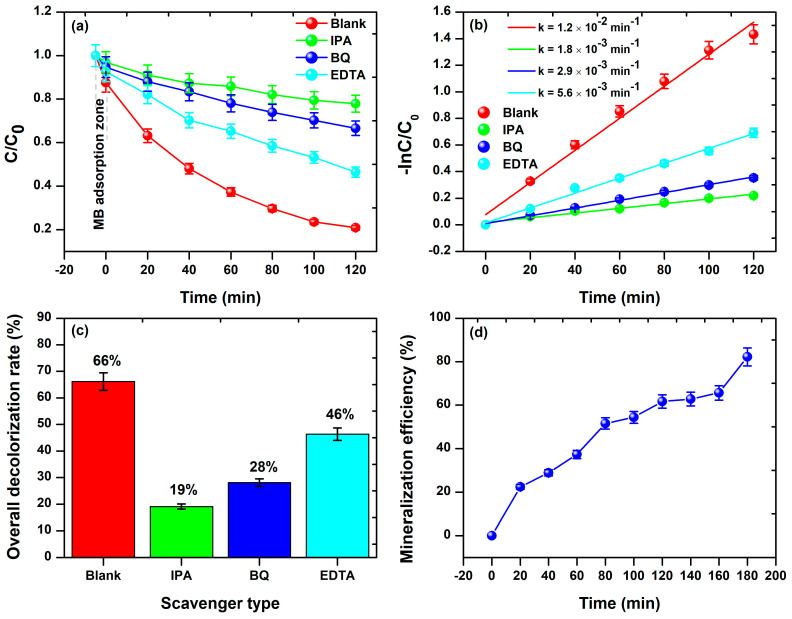
(**a**) The C/C_0_ factor, (**b**) First-order kinetics plots, and (**c**) Overall decolorization rate based on the elemental trapping experiment. (**d**) TOC removal efficiency of MB dye degradation was determined using the aliquots collected in the 1st degradation cycle.

We conducted a detailed investigation into the role and efficiency of chemical species generated during a photocatalytic experiment, employing an elemental trapping analysis, as illustrated in Figure 9. Following 120 min of visible light irradiation, the control experiment (Blank) exhibited the most effective degradation (Figure 9a). However, the introduction of IPA scavenger into the MB-BFDW solution significantly impacted the photocatalytic process, remarkably reducing the dye degradation potential (Figure 9b), followed by BQ (Figure 9c), and EDTA (Figure 9d). The assessment of UV-Vis absorption curves obtained from the elemental trapping analysis is depicted in Figure 9. As observed, both C/C_0_ factors (Figure 10a) and photocatalytic reaction rates (Figure 10b) were lower for solutions containing scavengers. For instance, the photocatalytic reaction rate for the control experiment was determined to be 1.2 × 10^−2^ min^−1^, whereas the lowest rate was observed for the experiment with IPA scavenger (1.8 × 10^−3^ min^−1^). Additionally, Figure 10c reveals that the incorporation of the scavengers led to a significant reduction in the overall degradation rates, explicitly, 47% (IPA), 38% (BQ), and 20% (EDTA), respectively. This indicates that the hierarchy of significance among the scavengers for the photocatalytic process is given as follows: IPA > BQ > EDTA. It is known that IPA, BQ, and EDTA function as scavengers for free OH• ions, O2−• anions, and holes (h^+^), respectively. Consequently, OH• and O2−• arise as the main chemical species involved in the degradation process of MB molecules in aqueous solutions containing BFD waste under visible light irradiation. Furthermore, the 1st photodegradation cycle achieved an 82% total organic carbon (TOC) removal (Figure 10d), confirming the degradation and mineralization of the hazardous organic molecules of MB dye through the action of OH• and O2−• chemical species.

### 3.3. Probable Mechanism of the Photocatalytic Activity of BFDW

**Figure 11:**
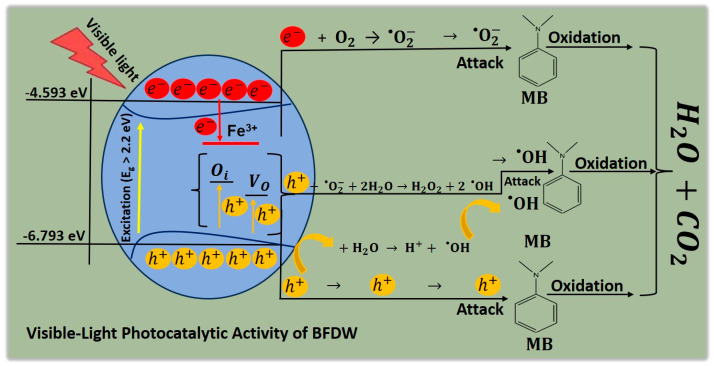
Probable mechanism of MB dye degradation mediated by the use of BFDW powder as a photocatalyst.

The probable mechanism of MB dye photodegradation mediated by the use of BFDW powder as a photocatalysts under visible light is schematized in Figure 11. Initially, the aqueous suspension containing the MB and BFDW particles undergoes an initial process of equilibration, including adsorption of oxygen, water, and MB molecules on the BFDW surface. Under visible light, BFDW absorbs energy to surpass its E_g_ = 2.2 eV, triggering redox reactions that lead to the mineralization of MB dye molecules. For this purpose, the electrons move from the VB to the CB, resulting in the creation of the charge carriers (e^−^/h^+^), as demonstrated in Equation (10) [126,127]. Quickly, the charge carriers arrive at the surface of BFDW, where they react with O_2_ and H_2_O molecules to give rise to the reactive chemical species O2−• and OH• (Equations (11) and (12)), respectively.

(10) $$BFDW+Visible\;Light\to BFDW\left(e_{CB}^{-}\right)+BFDW(h_{VB}^{+})$$

(11) BFDWeCB−+O2→BFDWO2−•

(12) BFDWhVB++H2O→H++OH•

Additionally, specific structural properties of BFDW can influence the photocatalytic process. As observed, O_i_ and V_O_ were the main structural defects found in the BFDW structure. These defects can trap holes in the optical bandgap of BFDW, which subsequently interact with O2−• and H_2_O to generate new chemical species like OH• and $H_{2}O_{2}$ (Equations (13) and (14)). Further, $H_{2}O_{2}$ can also be photocatalyzed by visible light, generating more OH• species (Equation (15)), introducing an additional mechanism for generating O2−• and OH• highly reactive chemical species that are responsible for the decolorization of MB dye molecules. Finally, these chemical species react with MB dye structure, generating simpler molecules such as H_2_O and CO_2_ [128,129,130] (Equations (16) and (17)), resulting in decolorization of the blue solution. Moreover, our elemental trapping analysis confirms that O2−• and OH• are the primary chemical species accountable for the degradation of MB molecules. Nevertheless, the existing literature establishes that holes alone can oxidize MB molecules, concurrently producing CO_2_ and H_2_O molecules (Equation (18)) [131,132,133,134].

(13) hOi/ZnO/α−Fe2O3++2H2O+O2−•→2OH•+H2O2

(14) hVO/ZnO/α−Fe2O3++2H2O+O2−•→2OH•+H2O2

(15) H2O2+visible light→2OH•

(16) O2−•+MB→CO2+H2O

(17) OH•+MB→CO2+H2O

(18) $$h^{+}+MB\to {CO}_{2}{+H}_{2}O$$

### 3.4. Comparing BFDW’s Photocatalytic Activity to Other Residues

The existing literature includes some research that investigated the efficiency of different wastes in removing dyes, including methylene blue, rhodamine, remazol black, methyl orange, and bromophenol blue, as shown in Table 4. Our comparative analysis was performed considering different experimental conditions e.g., pollutant type (PT), photocatalyst mass (PM), pollutant concentration (PC), reaction time (RT), irradiation source (IS), and photocatalytic performance (PP). As illustrated, there are significant variations in dye removal performance. Herein, BFD reached an efficiency of 74%, surpassing previous studies that reported lower values, e.g., 46% [135], 70% [136], 71% [41], and 72% [40,137]. Despite the utilization of shorter irradiation times in these previous studies, it is noteworthy that a UV irradiation source, which is considerably more energetic, was employed. This higher-energy UV source has the inherent capability to degrade organic dye molecules naturally. Furthermore, 10 mg was used in our experiment, which is notably lower than most values used in these studies (10–200 mg). Nevertheless, some other waste-based materials demonstrated comparable or superior photocatalytic performance at shorter irradiation times, e.g., 80% [138], 77% [139], and 74% [140], but also using a considerably large amount of the photocatalyst during the photocatalytic experiment. Hence, our BFD waste demonstrates photocatalytic performance comparable to other waste materials despite the use of a relatively low quantity of photocatalyst in the photocatalytic experiment. This underscores the promising potential of BFD waste as a photocatalyst, mainly considering its ability to be appropriately recycled and reused for up to 3 degradation cycles. This sustainability aspect further enhances the attractiveness of BFD waste as an effective and eco-friendly alternative for photocatalytic applications.

## 4. Conclusions

In summary, the comprehensive analysis of pure BFDW powder revealed its complex composition, structural characteristics, morphological properties, and potential for photocatalytic applications. The main crystalline phase found in BFDW was α-Fe_2_O_3_, comprising approximately 90% of the total composition, with minor quantities of CaO, K_2_O, ZnO, and MnO. The structural analysis confirmed the presence of α-Fe_2_O_3_ and ZnO as the main phases that may underlie the photocatalytic activity of BFDW. BFDW powder exhibited a porous surface with diverse shapes and arrangements, primarily composed of highly porous structures agglomerated and adorned with distinctive-shaped 10–30 nm nanoparticles. Chemical and thermal analyses highlighted the existence of surface-bound water molecules, carbonate molecules, and the thermal stability of BFDW up to 900 °C. Electrical conductivity analysis revealed charge transfer resistance values of 616.4 Ω and electrode resistance of 47.8 Ω. Mott–Schottky analysis identified α-Fe_2_O_3_ as an n-type semiconductor with a flat band potential of 0.181 V vs. Ag/AgCl and a donor density of 1.45 × 10^15^ cm^−3^. The optical bandgap and luminescence properties of BFDW powder were primarily attributed to α-Fe_2_O_3_ and ZnO phases. Moreover, BFDW exhibited weak ferromagnetic behavior ascribed to its structural defects and nanoparticles. A maximum photocatalytic efficiency of 74%, stable up to 3 photodegradation cycles, was demonstrated through the degradation of methylene blue (MB) dye under visible light. Elemental trapping experiments revealed the involvement of O2−• and OH• as the main chemical species responsible for MB dye degradation. In comparison with other waste materials, BFDW demonstrates competitive photocatalytic performance, highlighting its potential as an ecofriendly and effective alternative for photocatalytic applications. Therefore, our findings demonstrate the importance of BFDW’s unique physical properties in influencing its photocatalytic properties, paving the way for its sustainable utilization in affordable wastewater treatment processes.

## Figures and Tables

**Table 1:** Chemical composition of BFDW.

Sample	Structure	Composition (%)
BFDW	α-Fe_2_O_3_	94.30
	CaO	2.74
	K_2_O	1.6
	ZnO	0.73
	MnO	0.63

**Table 2:** Surface area, volume, and pore diameter data for blast furnace dust waste.

Parameter	Unit	Values
Surface area	m^2^ g^−1^	284
Pore volume	cm^3^ g^−1^	0.18
Average pore diameter	Å	15.27

**Table 3:** Defects assignments of BFD waste evaluated via photoluminescence.

Sample	Peak Position (nm)	Energy Level (eV)	Defect	References
BFWD	285	4.35	MBE	[95,96]
	302	4.11	MBE	[97]
	345	3.59	MBE, V_O_	[89]
	374	3.32	VB → CB	[90,98]
	389	3.19	NBE, $h^{+}$	[17,99]
	405	3.06	V_O_	[17,100]
	420	2.95	O_i_, V_O_	[91,92,93]
	468	2.65	V_O_	[84,94]

**Table 4:** Comparative analysis of the photocatalytic performance between BFD and other wastes under different experimental conditions.

Waste	PT	PM	PC	RT	IS	PP	Reference
	-	mg	mg∙L^−1^	min	-	%	-
BFD	methylene blue	10	10	180	Visible light	74	Here
Electric arc furnace dust	rhodamine B	150	1	140	UV light	70	[136]
Electric arc furnace dust	remazol black	120	40	150	UV light	72	[137]
Electric arc furnace dust	methylene blue	50	2,5	120	UV light	80	[138]
Chromite ore process waste	methylene blue	70	10	80	UV light	77	[139]
Turmeric leaves powder	methyl orange	10	10	120	Visible light	46	[135]
TiO_2_ supported on tar pitch and red mud	remazol black B	200	40	240	UV light	71	[41]
Biosilica from date palm biomass ash	bromophenol Blue	50	20	129	UV light	74	[140]
Metals-doped α-Fe_2_O_3_ prepared from BFD	methylene blue	50	10	150	UV light	72	[40]

## Data Availability

The data used to support the findings of this study are available from the corresponding author upon request.

## Supplementary Materials

The following supporting information can be downloaded at: https://www.mdpi.com/article/10.3390/ma17040818/s1.

## References

[1] Xiao X., Zhang S., Sher F., Chen J., Xin Y., You Z., Wen L., Hu M., Qiu G. (2021). A Review on Recycling and Reutilization of Blast Furnace Dust as a Secondary Resource. J. Sustain. Metall..

[2] Nayak N.P. (2022). Characterization of Blast Furnace Flue Dust—An Assessment for Its Utilization. Mater. Today Proc..

[3] Soria-Aguilar M.d.J., Martínez-Luévanos A., Sánchez-Castillo M.A., Carrillo-Pedroza F.R., Toro N., Narváez-García V.M. (2021). Removal of Pb(II) from Aqueous Solutions by Using Steelmaking Industry Wastes: Effect of Blast Furnace Dust’s Chemical Composition. Arab. J. Chem..

[4] Xu J., Wang N., Zhou Z., Chen M., Wang P. (2020). Experimental and Numerical Studies of the Gas-Molten Reduction Behavior of Blast Furnace Dust Particles during in-Flight Process. Powder Technol..

[5] Zhang Y., Li M., Huang W., Fan K., Li J., Zhong M., Li Z., Li C., Zhang Q. (2022). Research on Modified Blast Furnace Dust in Demulsification: The Synergistic Effect of Ferric Oxide, Hydrophobic Carbon, and Polysilicate. J. Air Waste Manag. Assoc..

[6] Xu J., Wang N., Chen M., Zhou Z., Yu H. (2020). Comparative Investigation on the Reduction Behavior of Blast Furnace Dust Particles during In-Flight Process in Hydrogen-Rich and Carbon Monoxide Atmospheres. Powder Technol..

[7] Das R., Mondal M.K., Pramanik S. (2023). Enhancement of Strength of Blast Furnace Flue Dust—Iron Oxide—Fly Ash Composite Briquette Using ANOVA-Based Mathematical Model. Ironmak. Steelmak..

[8] Qureshi A.A., Javed S., Javed H.M.A., Jamshaid M., Ali U., Akram M.A. (2022). Systematic Investigation of Structural, Morphological, Thermal, Optoelectronic, and Magnetic Properties of High-Purity Hematite/Magnetite Nanoparticles for Optoelectronics. Nanomaterials.

[9] Ashraf M., Khan I., Usman M., Khan A., Shah S.S., Khan A.Z., Saeed K., Yaseen M., Ehsan M.F., Tahir M.N. (2020). Hematite and Magnetite Nanostructures for Green and Sustainable Energy Harnessing and Environmental Pollution Control: A Review. Chem. Res. Toxicol..

[10] Mishra M., Chun D.-M. (2015). α-Fe_2_O_3_ as a Photocatalytic Material: A Review. Appl. Catal. A Gen..

[11] Kumar Y., Kumar R., Raizada P., Khan A.A.P., Singh A., Van Le Q., Nguyen V.-H., Selvasembian R., Thakur S., Singh P. (2022). Current Status of Hematite (α-Fe_2_O_3_) Based Z-Scheme Photocatalytic Systems for Environmental and Energy Applications. J. Environ. Chem. Eng..

[12] Liu T., Morelli M., Li Y., Sankir N.D., Sankir M. (2018). Hematite Materials for Solar-Driven Photoelectrochemical Cells. Photoelectrochemical Solar Cells.

[13] Katsuki T., Zahran Z.N., Tanaka K., Eo T., Mohamed E.A., Tsubonouchi Y., Berber M.R., Yagi M. (2021). Facile Fabrication of a Highly Crystalline and Well-Interconnected Hematite Nanoparticle Photoanode for Efficient Visible-Light-Driven Water Oxidation. ACS Appl. Mater. Interfaces.

[14] Hitam C.N.C., Jalil A.A. (2020). A Review on Exploration of Fe_2_O_3_ Photocatalyst towards Degradation of Dyes and Organic Contaminants. J. Environ. Manag..

[15] Fawzi Suleiman Khasawneh O., Palaniandy P. (2021). Removal of Organic Pollutants from Water by Fe_2_O_3_/TiO_2_ Based Photocatalytic Degradation: A Review. Environ. Technol. Innov..

[16] Xiao C., Li J., Zhang G. (2018). Synthesis of Stable Burger-like α-Fe_2_O_3_ Catalysts: Formation Mechanism and Excellent Photo-Fenton Catalytic Performance. J. Clean. Prod..

[17] Matos R.S., Monteiro M.D.S., Silva R.S., Macêdo M.A., Paz S.P.A., Angélica R.S., Oliveira R.M.P.B., Ferreira N.S. (2022). Novel Amapá Latex-Mediated Synthesis of Defective α-Fe_2_O_3_ Nanoparticles with Enhanced Ferromagnetism and Sunlight Photocatalytic Activity. Ceram. Int..

[18] Leonel A.G., Mansur A.A.P., Mansur H.S. (2021). Advanced Functional Nanostructures Based on Magnetic Iron Oxide Nanomaterials for Water Remediation: A Review. Water Res..

[19] Abdulkadir I., Abdallah H.M.I., Jonnalagadda S.B., Martincigh B.S. (2018). The Effect of Synthesis Method on the Structure, and Magnetic and Photocatalytic Properties of Hematite (α-Fe_2_O_3_) Nanoparticles—Research Article. S. Afr. J. Chem..

[20] Ma T., Zheng L., Zhao Y., Xu Y., Zhang J., Liu X. (2019). Highly Porous Double-Shelled Hollow Hematite Nanoparticles for Gas Sensing. ACS Appl. Nano Mater..

[21] Jung W.-S., Park S.-H., Kadam A.N., Kim H., Lee S.-W. (2020). Direct Hydrothermal Synthesis of Amine-Functionalized Cubic Hematite (C-Fe_2_O_3_) and Sonochemical Deposition of Nanosized Au for Its Application as a Visible-Light Photocatalyst. Dalton Trans..

[22] Lai F., Feng J., Heil T., Wang G.-C., Adler P., Antonietti M., Oschatz M. (2019). Strong Metal Oxide-Support Interactions in Carbon/Hematite Nanohybrids Activate Novel Energy Storage Modes for Ionic Liquid-Based Supercapacitors. Energy Storage Mater..

[23] Ikram H., Al Rashid A., Koç M. (2022). Synthesis and Characterization of Hematite (α-Fe_2_O_3_) Reinforced Polylactic Acid (PLA) Nanocomposites for Biomedical Applications. Compos. Part C Open Access.

[24] Lunin A.V., Lizunova A.A., Mochalova E.N., Yakovtseva M.N., Cherkasov V.R., Nikitin M.P., Kolychev E.L. (2020). Hematite Nanoparticles from Unexpected Reaction of Ferrihydrite with Concentrated Acids for Biomedical Applications. Molecules.

[25] Hao H., Sun D., Xu Y., Liu P., Zhang G., Sun Y., Gao D. (2016). Hematite Nanoplates: Controllable Synthesis, Gas Sensing, Photocatalytic and Magnetic Properties. J. Colloid Interface Sci..

[26] Su M., Pan Z., Chong Y., Ye C., Jin X., Wu Q., Hu Z., Ye D., Waterhouse G.I.N., Qiu Y. (2021). Boosting the Electrochemical Performance of Hematite Nanorods via Quenching-Induced Metal Single Atom Functionalization. J. Mater. Chem. A.

[27] Tofanello A., Shen S., de Souza F.L., Vayssieres L. (2020). Strategies to Improve the Photoelectrochemical Performance of Hematite Nanorod-Based Photoanodes. APL Mater..

[28] R Ochoa Díaz A.L.D. (2019). Electric Arc Furnace Slag and Blast Furnace Dust, Use for the Manufacture of Asphalt Concrete for Roads. Civ. Eng. Infrastruct. J..

[29] Ochoa R., López A., Grimaldo G. (2021). Behavior of the Dynamic Modulus and Fatigue in Asphalt Mixtures with Blast Oxygen Furnace Slag and Blast Furnace Dust. Period. Polytech. Civ. Eng..

[30] Xie B., Geng N., Yu Q., He D., Wang F., Liu T., Gao J., Ning P., Song X., Jia L. (2022). Removal of SO_2_ from Flue Gas Using Blast Furnace Dust as an Adsorbent. Environ. Sci. Pollut. Res..

[31] Bagatini M.C., Fernandes T., Silva R., Galvão D.F., Flores I.V. (2020). Mill Scale and Flue Dust Briquettes as Alternative Burden to Low Height Blast Furnaces. J. Clean. Prod..

[32] Zhang L., Gao Y., Yue Q., Zhang P., Wang Y., Gao B. (2020). Prepartion and Application of Novel Blast Furnace Dust Based Catalytic-Ceramic-Filler in Electrolysis Assisted Catalytic Micro-Electrolysis System for Ciprofloxacin Wastewater Treatment. J. Hazard. Mater..

[33] Guo J., Zhang Y., Wen H., Jia H., Wang J. (2023). A Novel Recycling Way of Blast Furnace Dust from Steelworks: Electrocoagulation Coupled Micro-Electrolysis System in Indigo Wastewater Treatment. Chemosphere.

[34] Kurniawan A., Sutiono H., Indraswati N., Ismadji S. (2012). Removal of Basic Dyes in Binary System by Adsorption Using Rarasaponin–Bentonite: Revisited of Extended Langmuir Model. Chem. Eng. J..

[35] Daâssi D., Frikha F., Zouari-Mechichi H., Belbahri L., Woodward S., Mechichi T. (2012). Application of Response Surface Methodology to Optimize Decolourization of Dyes by the Laccase-Mediator System. J. Environ. Manag..

[36] Lalnunhlimi S., Krishnaswamy V. (2016). Decolorization of Azo Dyes (Direct Blue 151 and Direct Red 31) by Moderately Alkaliphilic Bacterial Consortium. Braz. J. Microbiol..

[37] Ahmed S.N., Haider W. (2018). Heterogeneous Photocatalysis and Its Potential Applications in Water and Wastewater Treatment: A Review. Nanotechnology.

[38] Chen L., Chuang Y., Nguyen T.B., Chang J.-H., Lam S.S., Chen C.-W., Dong C.-D. (2020). Novel Molybdenum Disulfide Heterostructure Nanohybrids with Enhanced Visible-Light-Induced Photocatalytic Activity towards Organic Dyes. J. Alloys Compd..

[39] Chen L., Tsai M.-L., Chuang Y., Chen C.-W., Dong C.-D. (2022). Construction of Carbon Nanotubes Bridged MoS2/ZnO Z-Scheme Nanohybrid towards Enhanced Visible Light Driven Photocatalytic Water Disinfection and Antibacterial Activity. Carbon N. Y..

[40] Wu Z.-J., Wang L.-C., Gao Z.-F., Liu W.-M., Wu X.-R. (2016). Recycling Blast Furnace Dust into Metals (Al, Zn and Ti)-Doped Hematite with Enhanced Photocatalytic Activity. J. Environ. Chem. Eng..

[41] de O. Pereira L., de Moura S.G., Coelho G.C.M., Oliveira L.C.A., de Almeida E.T., Magalhães F. (2019). Magnetic Photocatalysts from Industrial Residues and TiO_2_ for the Degradation of Organic Contaminants. J. Environ. Chem. Eng..

[42] Chen L., Chen C.-W., Dong C.-D. (2021). Direct Z-Scheme Heterostructures Based on MoSSe Quantum Dots for Visible Light-Driven Photocatalytic Tetracycline Degradation. ACS Appl. Nano Mater..

[43] Chen L., Chen C.-W., Huang C.-P., Chuang Y., Nguyen T.-B., Dong C.-D. (2022). A Visible-Light Sensitive MoSSe Nanohybrid for the Photocatalytic Degradation of Tetracycline, Oxytetracycline, and Chlortetracycline. J. Colloid Interface Sci..

[44] Fiorenza R., Di Mauro A., Cantarella M., Iaria C., Scalisi E.M., Brundo M.V., Gulino A., Spitaleri L., Nicotra G., Dattilo S. (2020). Preferential Removal of Pesticides from Water by Molecular Imprinting on TiO_2_ Photocatalysts. Chem. Eng. J..

[45] Zeshan M., Bhatti I.A., Mohsin M., Iqbal M., Amjed N., Nisar J., AlMasoud N., Alomar T.S. (2022). Remediation of Pesticides Using TiO_2_ Based Photocatalytic Strategies: A Review. Chemosphere.

[46] Matos R.S., Attah-Baah J.M., Monteiro M.D.S., Costa B.F.O., Mâcedo M.A., Silva Junior R.S., da Fonseca Filho H.D., Oliveira R.M.P.B., Ferreira N.S. (2023). Effect of the Amapá-Latex Chelating Agent Contents on the Microstructure and Photocatalytic Properties of ZnO Nanoparticles. J. Mater. Res. Technol..

[47] Luo X., Wang C., Shi X., Li X., Wei C., Li M., Deng Z. (2022). Selective Separation of Zinc and Iron/Carbon from Blast Furnace Dust via a Hydrometallurgical Cooperative Leaching Method. Waste Manag..

[48] Yang X., Xie B., Wang F., Ning P., Li K., Jia L., Feng J., Xia F. (2023). Resource Utilization of Hazardous Solid Waste Blast Furnace Dust: Efficient Wet Desulfurization and Metal Recovery. Chemosphere.

[49] Geerdes M., Chaigneau R., Lingiardi O., Molenaar R., van Opbergen R., Sha Y., Warren J. (2020). Modern Blast Furnace Ironmaking: An Introduction.

[50] Ashrit S.S., Sarkar S., Singh R., Yadav S.Z., Chatti R.V. (2020). Characterization of Blast Furnace Flue Dust—A Multi Analytical Techniques Approach. Metall. Res. Technol..

[51] Trinkel V., Mallow O., Thaler C., Schenk J., Rechberger H., Fellner J. (2015). Behavior of Chromium, Nickel, Lead, Zinc, Cadmium, and Mercury in the Blast Furnace—A Critical Review of Literature Data and Plant Investigations. Ind. Eng. Chem. Res..

[52] Butt A.R., Butt I.A., Nazir A., Ikram M., Sadiq S., Rashid K., Shujah T., Ali S. (2015). Molecular Imaging of CaO Nanowhiskers in Living Organs. Nucleus.

[53] Ikram M., Muhammad Khan A., Haider A., Haider J., Naz S., Ul-Hamid A., Shahzadi A., Nabgan W., Shujah T., Shahzadi I. (2022). Facile Synthesis of La- and Chitosan-Doped CaO Nanoparticles and Their Evaluation for Catalytic and Antimicrobial Potential with Molecular Docking Studies. ACS Omega.

[54] Albayatı S.H.M., Deveci P. (2019). PH and GSH Dual Responsive Smart Silica Nanocarrier for Doxorubicin Delivery. Mater. Res. Express.

[55] Wang X.-M., Zeng Y.-N., Jiang L.-Q., Wang Y.-T., Li J.-G., Kang L.-L., Ji R., Gao D., Wang F.-P., Yu Q. (2022). Highly Stable NaFeO_2_-Fe_3_O_4_ Composite Catalyst from Blast Furnace Dust for Efficient Production of Biodiesel at Low Temperature. Ind. Crops Prod..

[56] Koodynska D., Ryczkowski J., Hubicki Z. (2008). FT-IR/PAS Studies of Chelates Adsorption on Anion Exchangers. Eur. Phys. J. Spec. Top..

[57] Ferreira N.S., Sasaki J.M., Silva JR R.S., Attah-Baah J.M., Macêdo M.A. (2021). Visible-Light-Responsive Photocatalytic Activity Significantly Enhanced by Active [V Zn + V O +] Defects in Self-Assembled ZnO Nanoparticles. Inorg. Chem..

[58] Helmi M., Hemmati A. (2021). Synthesis of Magnetically Solid Base Catalyst of NaOH/Chitosan-Fe_3_O_4_ for Biodiesel Production from Waste Cooking Oil: Optimization, Kinetics and Thermodynamic Studies. Energy Convers. Manag..

[59] Fang Y., Zou T., Liang X., Wang S., Liu X., Gao X., Zhang Z. (2017). Self-Assembly Synthesis and Properties of Microencapsulated n-Tetradecane Phase Change Materials with a Calcium Carbonate Shell for Cold Energy Storage. ACS Sustain. Chem. Eng..

[60] Yusuff A.S., Owolabi J.O. (2019). Synthesis and Characterization of Alumina Supported Coconut Chaff Catalyst for Biodiesel Production from Waste Frying Oil. S. Afr. J. Chem. Eng..

[61] Thipperudra A., Nagaraja N., PrashanthKumar M., Arunkumar B. (2020). DSC and FTIR Studies in Potassium, Strontium Doped Boro-Phosphate Glasses. Chem. Mater. Res..

[62] Paulson E., Jothibas M. (2021). Significance of Thermal Interfacing in Hematite (α-Fe_2_O_3_) Nanoparticles Synthesized by Sol-Gel Method and Its Characteristics Properties. Surf. Interfaces.

[63] Fouad D.E., Zhang C., Bi C., Chand K., Lv J. (2020). A Facile Approach towards Large-Scale Synthesis of Pristine Hematite Nanoparticles with Enhanced Photo/Catalytic Properties via Annealing of Iron Sulfate Precursor in Presence of Treated Egg Shell Wastes as Desulfurizer. Mater. Charact..

[64] Zhao D., Zhang J., Wang G., Conejo A.N., Xu R., Wang H., Zhong J. (2016). Structure Characteristics and Combustibility of Carbonaceous Materials from Blast Furnace Flue Dust. Appl. Therm. Eng..

[65] Martins F.M., Neto J.M.d.R., Cunha C.J. (2008). da Mineral Phases of Weathered and Recent Electric Arc Furnace Dust. J. Hazard. Mater..

[66] Robinson R. (2005). High Temperature Properties of By-Product Cold Bonded Pellets Containing Blast Furnace Flue Dust. Thermochim. Acta.

[67] Al-harahsheh M., Al-Nu’airat J., Al-Otoom A., Al-hammouri I., Al-jabali H., Al-zoubi M., Abu Al’asal S. (2019). Treatments of Electric Arc Furnace Dust and Halogenated Plastic Wastes: A Review. J. Environ. Chem. Eng..

[68] Oustadakis P., Tsakiridis P.E., Katsiapi A., Agatzini-Leonardou S. (2010). Hydrometallurgical Process for Zinc Recovery from Electric Arc Furnace Dust (EAFD). J. Hazard. Mater..

[69] Monazam E.R., Breault R.W., Siriwardane R. (2014). Reduction of Hematite (Fe_2_O_3_) to Wüstite (FeO) by Carbon Monoxide (CO) for Chemical Looping Combustion. Chem. Eng. J..

[70] Aldegs Y., Elbarghouthi M., Elsheikh A., Walker G. (2008). Effect of Solution PH, Ionic Strength, and Temperature on Adsorption Behavior of Reactive Dyes on Activated Carbon. Dye. Pigment..

[71] Nabil K., Abdelmonem N., Nogami M., Ismail I. (2020). Preparation of Composite Monolith Supercapacitor Electrode Made from Textile-Grade Polyacrylonitrile Fibers and Phenolic Resin. Materials.

[72] Basri N.H., Deraman M., Kanwal S., Talib I.A., Manjunatha J.G., Aziz A.A., Farma R. (2013). Supercapacitors Using Binderless Composite Monolith Electrodes from Carbon Nanotubes and Pre-Carbonized Biomass Residues. Biomass Bioenergy.

[73] Bhuyan M.A.H., Gebre R.K., Finnilä M.A.J., Illikainen M., Luukkonen T. (2022). Preparation of Filter by Alkali Activation of Blast Furnace Slag and Its Application for Dye Removal. J. Environ. Chem. Eng..

[74] Restrepo C.V., Villa C.C. (2021). Synthesis of Silver Nanoparticles, Influence of Capping Agents, and Dependence on Size and Shape: A Review. Environ. Nanotechnol. Monit. Manag..

[75] Kim D., Min K.-D., Lee J., Park J.H., Chun J.H. (2006). Influences of Surface Capping on Particle Size and Optical Characteristics of ZnS:Cu Nanocrystals. Mater. Sci. Eng. B.

[76] Kumar S.V., Ganesan S. (2011). Preparation and Characterization of Gold Nanoparticles with Different Capping Agents. Int. J. Green Nanotechnol..

[77] Parveen R., Datta A., Kumar Maiti P. (2021). Concentration of Capping Agent Controls Size Selection, Agglomeration and Antimicrobial Action of Silver Nanoparticles. J. Surf. Sci. Technol..

[78] Mizuno S., Yao H. (2021). On the Electronic Transitions of α-Fe_2_O_3_ Hematite Nanoparticles with Different Size and Morphology: Analysis by Simultaneous Deconvolution of UV–Vis Absorption and MCD Spectra. J. Magn. Magn. Mater..

[79] Mansour H., Omri K., Ammar S. (2019). Structural, Optical and Magnetic Properties of Cobalt Doped Hematite Nanoparticles. Chem. Phys..

[80] Pradhan G.K., Parida K.M. (2011). Fabrication, Growth Mechanism, and Characterization of α-Fe_2_O_3_ Nanorods. ACS Appl. Mater. Interfaces.

[81] Soares V.A., Xavier M.J.S., Rodrigues E.S., de Oliveira C.A., Farias P.M.A., Stingl A., Ferreira N.S., Silva M.S. (2020). Green Synthesis of ZnO Nanoparticles Using Whey as an Effective Chelating Agent. Mater. Lett..

[82] Ai M., Li X., Pan L., Xu X., Yang J., Zou J.-J., Zhang X. (2022). Surface States Modulation of Hematite Photoanodes for Enhancing Photoelectrochemical Catalysis. Chem. Eng. Sci..

[83] Popov N., Ristić M., Bošković M., Perović M., Musić S., Stanković D., Krehula S. (2022). Influence of Sn Doping on the Structural, Magnetic, Optical and Photocatalytic Properties of Hematite (α-Fe_2_O_3_) Nanoparticles. J. Phys. Chem. Solids.

[84] Rufus A., Sreeju N., Philip D. (2019). Size Tunable Biosynthesis and Luminescence Quenching of Nanostructured Hematite (α-Fe_2_O_3_) for Catalytic Degradation of Organic Pollutants. J. Phys. Chem. Solids.

[85] Hjiri M., Alonizan N.H., Althubayti M.M., Alshammari S., Besbes H., Aida M.S. (2019). Preparation and Photoluminescence of NiFe_2_O_4_ Nanoparticles. J. Mater. Sci. Mater. Electron..

[86] Sundara Selvam P.S., Govindan S., Perumal B., Kandan V. (2021). Screening of In Vitro Antibacterial Property of Hematite (α-Fe_2_O_3_) Nanoparticles: A Green Approach. Iran. J. Sci. Technol. Trans. A Sci..

[87] Kumar S., Kumar A., Malhotra T., Verma S. (2022). Characterization of Structural, Optical and Photocatalytic Properties of Silver Modified Hematite (α-FeO) Nanocatalyst. J. Alloys Compd..

[88] Rahmani M.B., Ghasemi E., Rezaii F. (2020). Synthesis of Wormlike α-Fe_2_O_3_ Nanostructure: Characterization and Antibacterial Application. J. Electron. Mater..

[89] Rada S., Culea E., Rada M. (2018). Novel ZrO2 Based Ceramics Stabilized by Fe_2_O_3_, SiO_2_ and Y_2_O_3_. Chem. Phys. Lett..

[90] Lassoued A., Dkhil B., Gadri A., Ammar S. (2017). Control of the Shape and Size of Iron Oxide (α-Fe_2_O_3_) Nanoparticles Synthesized through the Chemical Precipitation Method. Results Phys..

[91] Marzouk M.A., Abo-Naf S.M., Zayed H.A., Hassan N.S. (2016). Photoluminescence and Semiconducting Behavior of Fe, Co, Ni and Cu Implanted in Heavy Metal Oxide Glasses. J. Mater. Res. Technol..

[92] Fonseca Filho H.D., Pires M.P., Souza P.L., Matos R.S., Prioli R. (2022). Investigation of the Morphological and Fractal Behavior at Nanoscale of Patterning Lines by Scratching in an Atomic Force Microscope. Microsc. Res. Tech..

[93] Yang A., Yang Y., Zhang Z., Bao X., Yang R., Li S., Sun L. (2013). Photoluminescence and Defect Evolution of Nano-ZnO Thin Films at Low Temperature Annealing. Sci. China Technol. Sci..

[94] Ravichandran A.T., Karthick R. (2020). Enhanced Photoluminescence, Structural, Morphological and Antimicrobial Efficacy of Co-Doped ZnO Nanoparticles Prepared by Co-Precipitation Method. Results Mater..

[95] Justus J.S., Roy S.D.D., Raj A.M.E. (2016). Influence of Lanthanum Doping on the Structural and Optical Properties of Hematite Nanopowders. J. Appl. Sci. Eng. Methodol..

[96] Wang J., White W.B., Adair J.H. (2005). Optical Properties of Hydrothermally Synthesized Hematite Particulate Pigments. J. Am. Ceram. Soc..

[97] Wang J., Zhou Z., Xue J. (2006). Phase Transition, Ferroelectric Behaviors and Domain Structures of (Na_1/2_Bi_1/2_)_1−x_TiPb_x_O_3_ Thin Films. Acta Mater..

[98] Ling Y., Wheeler D.A., Zhang J.Z., Li Y. (2013). Optical Properties and Applications of Hematite (α-Fe_2_O_3_) Nanostructures. One-Dimensional Nanostructures.

[99] Fakhri A., Naji M., Nejad P.A. (2017). Adsorption and Photocatalysis Efficiency of Magnetite Quantum Dots Anchored Tin Dioxide Nanofibers for Removal of Mutagenic Compound: Toxicity Evaluation and Antibacterial Activity. J. Photochem. Photobiol. B Biol..

[100] Mathevula L.E., Noto L.L., Mothudi B.M., Chithambo M., Dhlamini M.S. (2017). Structural and Optical Properties of Sol-Gel Derived α-Fe_2_O_3_ Nanoparticles. J. Lumin..

[101] Tadic M., Trpkov D., Kopanja L., Vojnovic S., Panjan M. (2019). Hydrothermal Synthesis of Hematite (α-Fe_2_O_3_) Nanoparticle Forms: Synthesis Conditions, Structure, Particle Shape Analysis, Cytotoxicity and Magnetic Properties. J. Alloys Compd..

[102] Özdemir Ö., Dunlop D.J. (2014). Hysteresis and Coercivity of Hematite. J. Geophys. Res. Solid Earth.

[103] Smart T.J., Ping Y. (2017). Effect of Defects on the Small Polaron Formation and Transport Properties of Hematite from First-Principles Calculations. J. Phys. Condens. Matter.

[104] Vandenberghe R.E., Van San E., De Grave E., Da Costa G.M. (2001). About the Morin Transition in Hematite in Relation with Particle Size and Aluminium Substitution. Czechoslov. J. Phys..

[105] Issa B., Obaidat I., Albiss B., Haik Y. (2013). Magnetic Nanoparticles: Surface Effects and Properties Related to Biomedicine Applications. Int. J. Mol. Sci..

[106] Letcher T. (2022). Comprehensive Renewable Energy.

[107] Sarma S.K., Mohan R., Shukla A. (2020). Structural, Opto-Electronic and Photoelectrochemical Properties of Tin Doped Hematite Nanoparticles for Water Splitting. Mater. Sci. Semicond. Process..

[108] Niu Y., Zhou Y., Niu P., Shen H., Ma Y. (2019). Effects of Ti Doping on Hematite Photoanodes: More Surface States. J. Nanosci. Nanotechnol..

[109] She X., Zhang Z., Baek M., Yong K. (2018). Photoelectrochemical Enhancement of ZnO/BiVO_4_/ZnFe_2_O_4_/Rare Earth Oxide Hetero-Nanostructures. Appl. Surf. Sci..

[110] Lee S.F., Jimenez-Relinque E., Martinez I., Castellote M. (2023). Effects of Mott–Schottky Frequency Selection and Other Controlling Factors on Flat-Band Potential and Band-Edge Position Determination of TiO_2_. Catalysts.

[111] Lee S.F., Jimenez-Relinque E., Martinez I., Castellote M. (2022). Photoelectrochemical Global Approach to the Behaviour of Nanostructured Anatase under Different Irradiation Conditions. Catal. Today.

[112] Li L., Liu C., Qiu Y., Mitsuzak N., Chen Z. (2017). The Influence of the Hydrothermal Temperature and Time on Morphology and Photoelectrochemical Response of α-Fe_2_O_3_ Photoanode. J. Alloys Compd..

[113] Wodka D., Socha R.P., Bielańska E., Elżbieciak-Wodka M., Nowak P., Warszyński P. (2014). Photocatalytic Activity of Titanium Dioxide Modified by Fe_2_O_3_ Nanoparticles. Appl. Surf. Sci..

[114] Chen H., Zhao J., Dai G. (2011). Silkworm Exuviae—A New Non-Conventional and Low-Cost Adsorbent for Removal of Methylene Blue from Aqueous Solutions. J. Hazard. Mater..

[115] Falletta E., Galloni M.G., Mila N., bin Roslan M.N., Abd Ghani N., Cerrato G., Giordana A., Magni M., Spriano S., Boffito D.C. (2023). Fast and Efficient Piezo-Photocatalytic Mineralization of Ibuprofen by BiOBr Nanosheets under Solar Light Irradiation. ACS Photonics.

[116] Kumar A., Raizada P., Hosseini-Bandegharaei A., Thakur V.K., Nguyen V.-H., Singh P. (2021). C-, N-Vacancy Defect Engineered Polymeric Carbon Nitride towards Photocatalysis: Viewpoints and Challenges. J. Mater. Chem. A.

[117] Ozin G.A. (2014). Nanochemistry Views.

[118] Alshehri A.A., Malik M.A. (2019). Biogenic Fabrication of ZnO Nanoparticles Using Trigonella Foenum-Graecum (Fenugreek) for Proficient Photocatalytic Degradation of Methylene Blue under UV Irradiation. J. Mater. Sci. Mater. Electron..

[119] Alharbi A., Abdelrahman E.A. (2020). Efficient Photocatalytic Degradation of Malachite Green Dye Using Facilely Synthesized Hematite Nanoparticles from Egyptian Insecticide Cans. Spectrochim. Acta Part A Mol. Biomol. Spectrosc..

[120] Ali H.R., Nassar H.N., El-Gendy N.S. (2017). Green Synthesis of α-Fe_2_O_3_ Using Citrus Reticulum Peels Extract and Water Decontamination from Different Organic Pollutants. Energy Sources Part A Recover. Util. Environ. Eff..

[121] Guo S., Hu Z., Zhen M., Gu B., Shen B., Dong F. (2020). Insights for Optimum Cation Defects in Photocatalysis: A Case Study of Hematite Nanostructures. Appl. Catal. B Environ..

[122] Feng H., Wang Y., Wang C., Diao F., Zhu W., Mu P., Yuan L., Zhou G., Rosei F. (2016). Defect-Induced Enhanced Photocatalytic Activities of Reduced α-Fe_2_O_3_ Nanoblades. Nanotechnology.

[123] Li L., Yan J., Wang T., Zhao Z.-J., Zhang J., Gong J., Guan N. (2015). Sub-10 Nm Rutile Titanium Dioxide Nanoparticles for Efficient Visible-Light-Driven Photocatalytic Hydrogen Production. Nat. Commun..

[124] Wang Y., Cai J., Wu M., Chen J., Zhao W., Tian Y., Ding T., Zhang J., Jiang Z., Li X. (2018). Rational Construction of Oxygen Vacancies onto Tungsten Trioxide to Improve Visible Light Photocatalytic Water Oxidation Reaction. Appl. Catal. B Environ..

[125] Li H., Li J., Ai Z., Jia F., Zhang L. (2018). Oxygen Vacancy-Mediated Photocatalysis of BiOCl: Reactivity, Selectivity, and Perspectives. Angew. Chem. Int. Ed..

[126] Haseena S., Shanavas S., Ahamad T., Alshehri S.M., Baskaran P., Duraimurugan J., Acevedo R., Khan M.A.M., Anbarasan P.M., Jayamani N. (2021). Investigation on Photocatalytic Activity of Bio-Treated α-Fe_2_O_3_ Nanoparticles Using Phyllanthus Niruri and Moringa Stenopetala Leaf Extract against Methylene Blue and Phenol Molecules: Kinetics, Mechanism and Stability. J. Environ. Chem. Eng..

[127] Weldegebrieal G.K., Sibhatu A.K. (2021). Photocatalytic Activity of Biosynthesized α-Fe_2_O_3_ Nanoparticles for the Degradation of Methylene Blue and Methyl Orange Dyes. Optik.

[128] Maji S.K., Jana A. (2017). Two-Dimensional Nanohybrid (RGS@AuNPs) as an Effective Catalyst for the Reduction of 4-Nitrophenol and Photo-Degradation of Methylene Blue Dye. New J. Chem..

[129] Lebron Y.A.R., Moreira V.R., Santos L.V.S., Jacob R.S. (2018). Remediation of Methylene Blue from Aqueous Solution by Chlorella Pyrenoidosa and Spirulina Maxima Biosorption: Equilibrium, Kinetics, Thermodynamics and Optimization Studies. J. Environ. Chem. Eng..

[130] Baruah R., Yadav A., Das A.M. (2021). Livistona Jekinsiana Fabricated ZnO Nanoparticles and Their Detrimental Effect towards Anthropogenic Organic Pollutants and Human Pathogenic Bacteria. Spectrochim. Acta Part A Mol. Biomol. Spectrosc..

[131] Guo M.Y., Ng A.M.C., Liu F., Djurišić A.B., Chan W.K. (2011). Photocatalytic Activity of Metal Oxides—The Role of Holes and OH Radicals. Appl. Catal. B Environ..

[132] Liu D., Tian R., Wang J., Nie E., Piao X., Li X., Sun Z. (2017). Photoelectrocatalytic Degradation of Methylene Blue Using F Doped TiO_2_ Photoelectrode under Visible Light Irradiation. Chemosphere.

[133] Geng K., Wu Y., Jiang G., Liu K., Jiang L. (2018). RuC@g-C_3_N_4_(H+)/TiO_2_ Visible Active Photocatalyst: Facile Fabrication and Z-Scheme Carrier Transfer Mechanism. Mol. Catal..

[134] Ghanei-Zare S., Moghadasi M., Khajavian R., Akbarzadeh-T N., Mirzaei M. (2023). A Metal-Organic Framework-Derived CuO Microrods for Fast Photocatalytic Degradation of Methylene Blue. J. Mol. Struct..

[135] Kumar A., Luxmi V. (2021). Development of an Efficient Eco-Friendly Photo-Catalyst Using Agro-Waste Turmeric Leaves and Its Characterizations. Optik.

[136] Sapiña M., Jimenez-Relinque E., Castellote M. (2014). Turning Waste into Valuable Resource: Potential of Electric Arc Furnace Dust as Photocatalytic Material. Environ. Sci. Pollut. Res..

[137] Almeida M.M., Saczk A.A., Felix F.d.S., Penido E.S., Santos T.A.R., de Souza Teixeira A., Magalhães F. (2023). Characterization of Electric Arc Furnace Dust and Its Application in Photocatalytic Reactions to Degrade Organic Contaminants in Synthetic and Real Samples. J. Photochem. Photobiol. A Chem..

[138] Alcaraz L., Urbieta A., Rabanal M.E., Fernández P., López F.A. (2020). Photocatalytic Activity of Electric-Arc Furnace Flue Dusts. J. Mater. Res. Technol..

[139] Zhang D., Zhang X., Zhang Z., Zhang X., Qiu F., Liu Z., Li W. (2022). Treatment of Methylene Blue Wastewater with Nano-PbCrO_4_ Photocatalyst Prepared from Chromite Ore Processing Residue. J. Clean. Prod..

[140] Elanthikkal S., Mohamed H.H., Alomair N.A. (2023). Extraction of Biosilica from Date Palm Biomass Ash and Its Application in Photocatalysis. Arab. J. Chem..

