# Supplementary material for: Associating Physical and Photocatalytic Properties of Recyclable and Reusable Blast Furnace Dust Waste
Source: Materials (Basel). 2024 Feb 8;17(4):818. doi: 10.3390/ma17040818 (PMC10889973; doi:10.3390/ma17040818)
Supplement: Supplementary file 1 [file materials-17-00818-s001.zip › materials-2814653-supplementary.pdf]

Supporting information

Associating Physical and Photocatalytic Properties of Recyclable and Reusable Blast Furnace Dust Waste

Nayane O. Chaves[1], Lucas S. Lima[2], Michael D. S. Monteiro[2], Raimundo A. L. Sobrinho[3], Nilson S. Ferreira[4], Glenda Q. Ramos[5], Henrique D. F. Filho[6], Rosane M. P. B. Oliveira[1], and Robert S. Matos[7*]

[1] Postgraduate Program in Materials Science and Engineering (P2CEM), Federal University of Sergipe, São Cristovão 49.100-000, SE, Brazil; nayaneochaves@academico.ufs.br; rosaneboliveira@academico.ufs.br
[2] Laboratory of Corrosion and Nanotechnology (LCNT), Federal University of Sergipe, 49100-000, São Cristovão 49.100-000, SE, Brazil; lsl.lucas@hotmail.com, michaelquimica96@gmail.com
[3] Department of Chemical Engineering, State University of Santa Cruz, Rod. Jorge Amado, Km 16 - Salobrinho, Ilhéus, 45.662-900, BA, Brazil; ralsobrinho@uesc.br
[4] Department of Physics, Federal University of Sergipe, 49.100-000, São Cristovão, SE, Brazil; nilson@academico.ufs.br
[5] Centro Multiusuário para Análise de Fenômenos Biomédicos da Universidade do Estado do Amazonas, Universidade Do Estado do Amazonas, Manaus 69.410-000, AM, Brazil; gq.ramos@hotmail.com
[6] Federal University of Amazonas-UFAM, Laboratory of Synthesis of Nanomaterials and Nanoscopy (LSNN), Physics Department, Manaus 69.077-000, AM, Brazil; hdffilho@ufam.edu.br
[7] Amazonian Materials Group, Federal University of Amapá (UNIFAP), Macapá 68.911-477, AP, Brazil.; robert_fisic@unifap.br

* Correspondence: Robert S. Matos, Amazonian Materials Group, Federal University of Amapá (UNIFAP), Macapá 68.911-477, AP, Brazil. email: robert_fisic@unifap.br

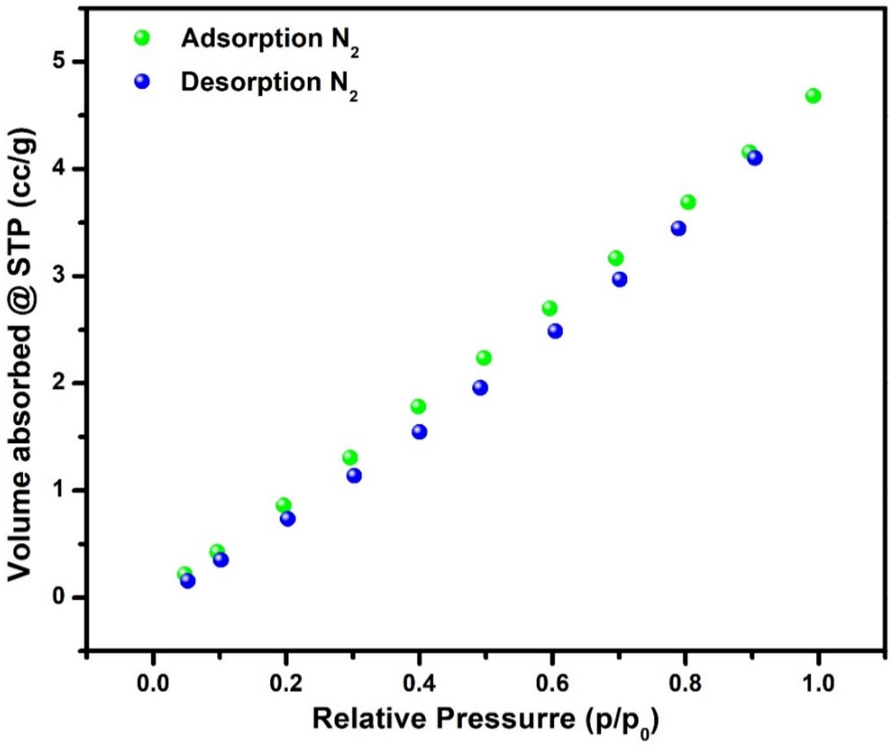

Figure S1. Adsorption–desorption isotherms of BFD waste.

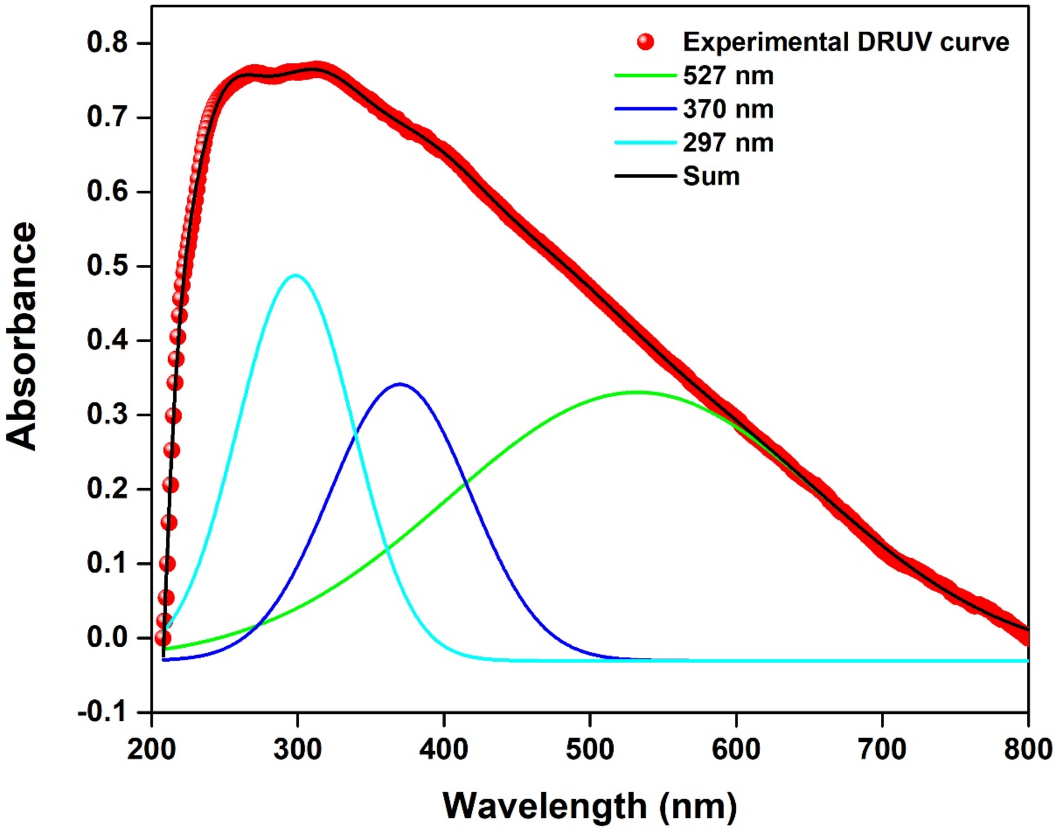

Figure S2. Deconvoluted DRUV spectrum of BFD waste.

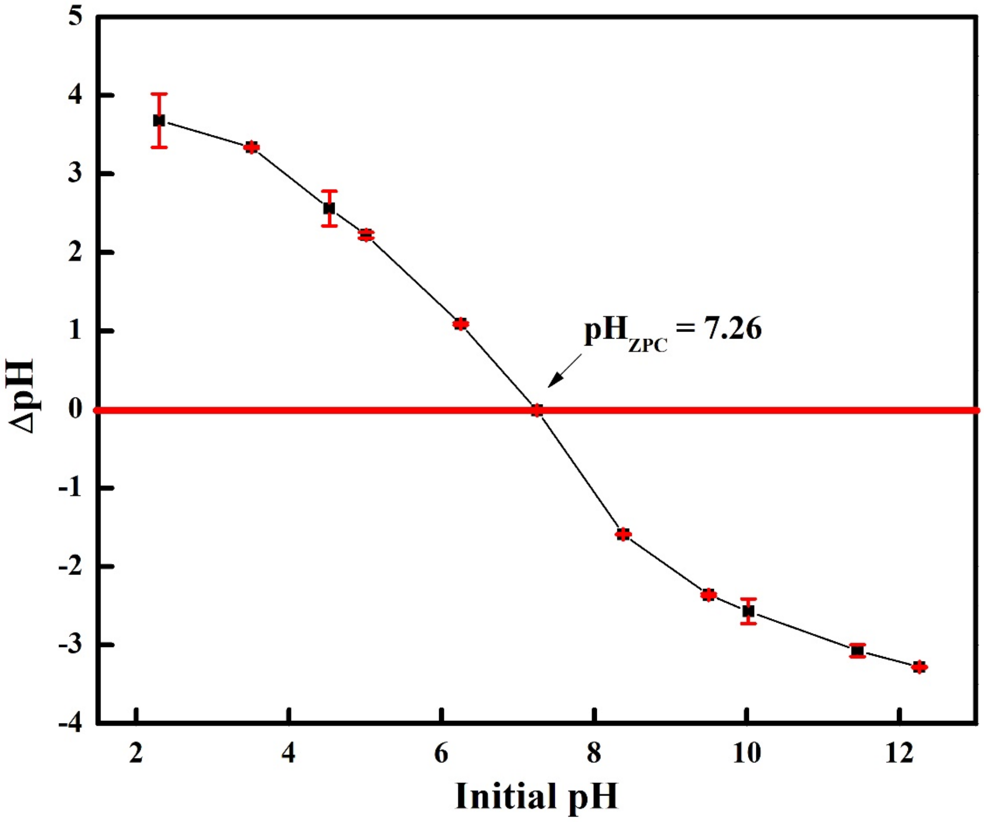

Figure S3. Point of zero charge of blast furnace dust waste.

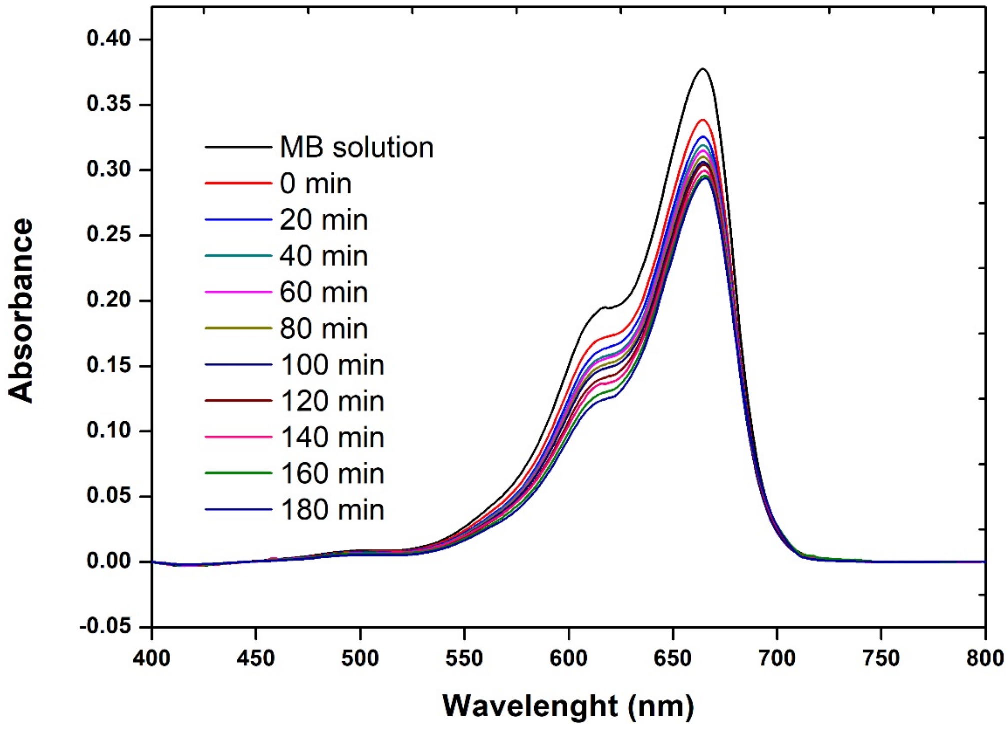

Figure S4. UV-vis absorption spectra of MB degradation in aqueous solutions containing BFDW in the dark.
